# A Connectomic Hypothesis for the Hominization of the Brain

**DOI:** 10.1093/cercor/bhaa365

**Published:** 2020-12-26

**Authors:** Jean-Pierre Changeux, Alexandros Goulas, Claus C Hilgetag

**Affiliations:** 1 CNRS UMR 3571, Institut Pasteur, 75724 Paris, France; 2 Communications Cellulaires, Collège de France, 75005 Paris, France; 3 Institute of Computational Neuroscience, University Medical Center Eppendorf, Hamburg University, 20246 Hamburg, Germany; 4 Department of Health Sciences, Boston University, Boston, MA 02115, USA

**Keywords:** brain hominization, brain phenotype, connectomic fundamentals, human genome

## Abstract

Cognitive abilities of the human brain, including language, have expanded dramatically in the course of our recent evolution from nonhuman primates, despite only minor apparent changes at the gene level. The hypothesis we propose for this paradox relies upon fundamental features of human brain connectivity, which contribute to a characteristic anatomical, functional, and computational neural phenotype, offering a parsimonious framework for connectomic changes taking place upon the human-specific evolution of the genome. Many human connectomic features might be accounted for by substantially increased brain size within the global neural architecture of the primate brain, resulting in a larger number of neurons and areas and the sparsification, increased modularity, and laminar differentiation of cortical connections. The combination of these features with the developmental expansion of upper cortical layers, prolonged postnatal brain development, and multiplied nongenetic interactions with the physical, social, and cultural environment gives rise to categorically human-specific cognitive abilities including the recursivity of language. Thus, a small set of genetic regulatory events affecting quantitative gene expression may plausibly account for the origins of human brain connectivity and cognition.

## Introduction: The Hominization of the Brain

### The Rise of Human Cognitive Abilities

A relatively short evolutionary period, of less than 2 million years, has resulted in major changes in the neural organization of the human brain, leading to a tremendous expansion of its cognitive capacities. These include, among others, a very large long-term memory storage capacity, enhanced working memory and conscious processing, rational thinking, cognitive flexibility, and self-awareness (Passingham 2008; Lagercrantz et al. 2010). They also concern unique social and cultural abilities including a theory of mind and, most distinctively, language, writing, enhanced command of tools, or pursuit of beauty (Vince 2019). While several hypotheses have been put forward to explain this astonishing cognitive evolution (Striedter 2005; Passingham 2008; Berwick and Chomsky 2016; Herculano-Houzel 2016; Ardesch et al. 2019), the question remains: how did such substantial, qualitative changes arise so rapidly in the brain as a consequence of apparently only few genetic differences between humans and other primates? The present hypothesis intends to find a minimal set of principles that allow us to explain uniquely human brain architecture in terms of a characteristic neuronal network—connectomic—organization as an intermediate anatomical, computational, and functional phenotype between the genome and the cognitive levels. The hypothesis is based on recent comparative data on brain connectomics and findings from network neuroscience that can illustrate how the evolution of human brain neuronal architecture—affecting mainly its microscopic organization—had major consequences on large-scale network organization and computations and, ultimately, human cognition, language, and culture.

Specifically, our hypothesis is based upon a set of “biological premises” relevant to the recent evolution of the human brain that may be summarized as follows.

1. In less than a few million years, major changes in the neural organization of the brain from the most recent ancestors of man up to *Homo sapiens* led to a tremendous expansion of its cognitive abilities including, among others, a very large long-term memory storage capacity, conscious processing and self-awareness, rational thinking, theory of mind, and, most distinctively, language (Striedter 2005; Passingham 2008; Berwick and Chomsky 2016).
2. These changes arise in the brain as a consequence of apparently only few genetic regulatory events differentiating between humans and other primates (Geschwind and Rakic 2013; Somel et al. 2013; Pääbo 2014; Vallender 2014)—as also illustrated by parallel RNA and DNA characterization of neuronal cell types in the human brain, indicating not only some patterns of development that we share with mice but also some aspects of interneuron development that are not observed in rodents (Huang et al. 2020). These events take place within the framework of a common brain organization (Rakic 2009; Arcaro and Livingstone 2017) shared among the primate ancestors of modern humans (Changeux 2017; Fishbein et al. 2020) and already structured on the basis of a rich set of genetic components—or “genetic envelope.”
3. The human brain contains vastly more neurons than that of other primates (Herculano-Houzel 2009). The increase appears particularly pronounced for the cerebral cortex where it results from an expansion of the number of cortical columns (Rakic 2009).
4. Correlatively, the number of cortical areas increases (Northcutt and Kaas 1995; Glasser et al. 2016).
5. The number of nested levels of organization of neuronal brain architecture and of the brain’s connectome scales up (Bassett et al. 2010) accompanied in particular by a nonlinear increase of white matter (i.e., long-range axonal connectivity) (Zilles 2005).
6. The core–periphery network architecture of the primate brain develops in humans to the benefit to the core long-range connectivity of the “global neuronal workspace” (Dehaene and Changeux 2011).
7. Meanwhile, in the cerebral cortex, a shift of cortical layer reafference from lower to upper layers selectively takes place in humans (Goulas et al. 2018).
8. A unique feature of the human brain is the extension of its postnatal development for up to 15 years and even longer during which the size of the brain increases ca. 5-fold. Considerable epigenetic processes of synapse selection (Changeux et al. 1973; Rakic 1976; Shatz and Stryker 1978; Purves and Lichtman 1980) and connectomic reorganization take place during this period. As a consequence, a net loss of the total number of synapses formed occurs late in childhood (Huttenlocher and Dabholkar 1997), but see Rakic et al. (1994). Last, a striking cultural diversification of brain connectivity develops between distinct social groups (Friederici 2017).

The present “connectomic hypothesis” intends, conceptually, to find a minimal set of principles that allow us to understand uniquely human brain architecture in terms of a characteristic neuronal network—connectomic—organization as an anatomical and functional phenotype linking the genome and the cognitive levels with major consequences on large-scale network organization and computations of the brain and, ultimately, human cognition, language, and culture in the course of its epigenetic postnatal complexification. The present hypothesis attempts to capture, first, the consequence of the absolute increase of brain size and number of neurons within the hominin lineage, and, second, the deviations from proportional scaling relationships that impose constraints upon human brain connectivity. These unique features would arise, for instance, from self-organization processes, including neuromodulatory mechanisms (Changeux 2005; Bargmann 2012; Changeux and Christopoulos 2017) together with intrinsic or environmentally elicited changes of the developing multilevel connectomic architecture of the human brain associated, in particular, with its exceptionally long postnatal epigenetic maturation.

Many aspects of this hypothesis are necessarily speculative at this point, since much information on human brain connectivity is currently derived from indirect approaches, for instance, by diffusion imaging, or extrapolation from mammalian animal models. Therefore, we here present a “working hypothesis” that needs to be substantiated by further empirical and computational studies. Last but not least, within the framework of the identification of the genetic regulatory events engaged in human brain evolution, our approach may be seen as some kind of reverse engineering in an attempt to infer the minimum number of “connectomic fundamentals” that parsimoniously account for the intrinsic evolution of the human brain connectome and the *H. sapiens*-specific, genetic regulatory events that determined them**.**

### The Case of Language

In this perspective, we examine the connectomic features of the human brain underlying its cognitive expansion, primarily focusing on language (Kuhl 2000; Friederici 2017), aware of the considerable literature on the topic and of the engagement of a multifaceted and highly specialized network of interlocking systems. Summarizing empirical approaches, Fitch (2017) has delineated some basic “derived components of language” that are unique to humans. A first component is “phonology,” the ability to acquire a basic lexicon, including symbols, that maps signals to concepts and dramatically develops in humans (Cheney and Seyfarth 1990; Savage-Rumbaugh et al. 1993; Kaminski 2004; Pilley and Reid 2011). Generally, with hominization occurs a remarkable increase and stabilization in representational capacity, including abstract symbolic and hierarchical representations, particularly by means of language. A second important component is the unique ability to produce an unlimited variety of linear signal strings, which communicate complex semantic messages in a recursive and hierarchical manner that is referred to as “dendrophilia” (Fitch 2017) or “merge” (Chomsky 1957, 2017; Berwick and Chomsky 2016; Friederici 2017) and includes conceptual blending (Fauconnier and Turner 2003). Third, “theory of mind” is the ability to represent the “representations of others’ thoughts” (Premack and Woodruff 1978; Bräuer et al. 2007; Penn and Povinelli 2007; Petanjek et al. 2019). It develops during the second year of life in humans (Lagercrantz et al. 2010; Kuhl 2011). In addition, synaptic reorganization still occurs up to the third decade within prefrontal cortex neurons (Petanjek et al. 2011). All these processes appear fundamental for the development of dendrophilia (Novack and Waxman 2020). Another human-specific trait is an exceptional proclivity to communicate socially or “glossogeny” (Coupland 2009; Fitch 2017), which is of special importance for cultural aspects of language and their diversity (Hurford 1990). It is associated with the prolonged postnatal synaptic epigenesis of the human infant and the ability to share culturally acquired knowledge with close kin, through teaching or “pedagogy” (Premack and Woodruff 1978; Premack and Premack 2003; Laland 2017).

In roughly the past 2 million years, most of the basic steps of human language acquisition and the formation of their neuronal bases have occurred (Fitch 2017, 2020). Here, we examine potential correspondence of the evolution of language functions to that of specific features of human brain connectivity.

## Human Brain-Specific Traits at the Genome Level

### Uniformity of Mammalian Genomes

The full-genome sequences now available for many animal species (mouse, monkey, chimpanzee, humans, and fossil human ancestors) are striking in their relative uniformity. The haploid human genome comprises no more than 20 000–25 000 gene-coding sequences (only 1.2% of the human genome). This number does not vary significantly from mouse to humans. Available comparative genomic data unambiguously show that the increase of brain anatomical and functional complexity does not reflect a parallel increase in genome complexity, in particular at the most recent stages of hominization. Examination of the evolution of protein-coding genes specifically expressed in different tissues of the human body (Dumas et al. 2019; Sjöstedt et al. 2020) further reveals that brain protein-coding genes involved in the neural substructures and synaptic organization were found more conserved than genes related to other parts of the body, testis being the most divergent. Moreover, the transcriptome of the diverse cortical cell type looks remarkably similar from mice to humans (Hodge et al. 2019). This general uniformity of mammalian coding genomes and of their transcriptional expression illustrates an astonishing “evolutionary parsimony” of genetic information (Changeux 1983, 2017).

### Differences in Gene Regulation

Nevertheless, several laboratories have tentatively identified sets of structural genes as plausible genetic events that separate humans from nonhuman primates (Ko et al. 2011; Geschwind and Rakic 2013; Somel et al. 2013; Pääbo 2014; Vallender 2014; Changeux 2017; Dumas et al. 2019), even though many of them might be neutral. Most of these genes are included among the hundreds (up to 500?) of those which mutation causes predisposition to autism-ASD or schizophrenia (Bourgeron 2015) and plausibly may represent suitable candidates for the evolution of human social cognition (Enard 2016). They are mostly involved in the general control of brain growth and neuronal number, neuronal maturation, and neurite outgrowth, for example, affecting brain size, cell division, growth arrest, nerve cell maturation, and DNA damage. Others are directly associated with neuronal aspects, such as transporters or neurotransmitter receptors, further with neurite outgrowth and synapse selection in the mammalian brain and more specifically with the extended synaptic development in the prefrontal cortex that distinguished human from rhesus monkey and chimpanzees (see genetic data of Liu et al. 2012). Last but not least, some genes have been directly related to language and speech, such as forkhead fox P2 (FOXP2), but nonetheless cannot be simply dubbed “language genes,” as most of them are already part of the large ensemble of genetic determinants that specifies the primate brain organization. There is no apparent “smoking gun” of structural gene differences that can be linked to cognitive abilities proper to humans, particularly language. The most likely possibility is differences in gene regulation. Along these lines, gene duplications (Ohno 1999) have been shown to occur in human lineage and for some of them exclusively in humans. Several of them have critical impact on the development of the cerebral cortex (Suzuki 2020). Among them is NOTCH2NL, which displays a copy number increase—up to 4—uniquely in humans (Duan et al. 2004; Fiddes et al. 2018; Florio et al. 2018; Suzuki et al. 2018). In addition, other genes regulating corticogenesis identified so far show gene duplications uniquely in humans, such as SRGAP2 (Charrier et al. 2012; Fossati et al. 2016) and ARHGAP11B (Florio et al. 2015, 2016; Kalebic et al. 2018). In sum, the copy numbers of NOTCH2, SRGAP2, and ARHGAP11 are increased specifically in humans, suggesting that their duplications had occurred in the human lineage after the last common ancestor with the chimpanzee (Suzuki 2020). MCPH1 and 5 are known to regulate the number of neuroblast symmetric divisions and control brain size (Dediu and Ladd 2007). Remarkably, the overexpression of human MCPH1 in transgenic rhesus monkeys led to an apparent delay in neuronal maturation and myelination as well as increase in relative gray matter volume and working memory (Shi et al. 2019). In a general manner, copy number variants (CNVs) appear as plausible targets of a positive selection engaged in the humanization of the brain (Hsieh et al. 2019).

Moreover, the important but largely unexplored vast noncoding regions of the human genome—its “dark matter”—are known to include point mutations, rearrangements, transposable element movements, and other changes that are absent in other close mammalian species (The Chimpanzee Sequencing and Analysis Consortium 2005). Some regions exhibit accelerated evolution (McLean et al. 2011; Holloway et al. 2016) together with changes in DNA regulatory sequences (Weyer and Pääbo 2016). New approaches are needed to identify the actual genetic regulatory events that are likely to have caused the fast increase in brain complexity during hominization (Mozzi et al. 2017).

### Gene Networks Underlying Brain Architecture and Connections

The central dogma of genetics, that one gene encodes one protein which itself encodes one phenotype at the organism level, breaks down for complex functions, particularly the cognitive abilities of the brain (Uttal 2001). Instead, one finds that “gene networks” encode neuronal networks and resulting behavioral phenotypes with the mobilization of multiple transcriptional and post-transcriptional events. This perspective advocates a radical change in the reductionist approach from higher brain functions to genes (Greenspan 2009; Changeux 2017). For instance, according to the “omnigenic” concept (Boyle et al. 2017), the heritability of complex traits of disease (or in our case, the connectome) is spread broadly across the genome (Loh et al. 2015; Shi et al. 2016), which implies that a substantial fraction of all genes contributes to it. The genes networks would then include, in addition to brain-specific “core” genes and pathways, abundant “peripheral” genes, all of them being highly interconnected, particularly at the long-range level.

A detailed mechanism for cooperative relationships among gene expression data based on transcription factor (TF) interactions was further proposed (Tsigelny et al. 2013). The approach, documented with genome-wide expression data, revealed evolutionary changes in TF networks from *Caenorhabditis elegans* (Hobert and Kratsios 2019) to macaque, chimpanzee, and humans (Mozzi et al. 2017; Berto and Nowick 2018). These studies illustrate the pleiotropic effect of genetic regulatory events on the brain connectome and the need of radically new approaches to identify the set of genetic regulatory events that specify what we refer to as “connectomic fundamentals,” which are actually engaged in the hominization of the brain. Our reverse engineering attempt is a step in this direction.

## Increased Brain Size, Number, and Diversity of Cortical Areas

### Increased Number of Cortical Neurons

The human brain contains vastly more neurons than that of other primates, at least 87 billion versus 6.4 billion in the macaque or 3.3 billion in the squirrel monkey brain (Herculano-Houzel 2009). This expansion accompanies the prolonged prenatal development of the human brain relative to other primates: chimpanzees 35 weeks, gorilla and orangutan 37 weeks, 38 weeks for humans (Finlay and Darlington 1995; Darlington et al. 1999; Jukic et al. 2013), which is also associated with an extraordinary gyrification of the cerebral cortex in humans (Rash et al. 2019).

Organoid studies in chimpanzees and humans (Mora-Bermúdez et al. 2016; Marchetto et al. 2019) further reveal that a notable difference between the two species is the length of S-phase metaphase in the mitosis of neural progenitors, which was found to be nearly 5 h longer in the human (17.5 h) than the chimpanzee (12.8 h). These observations are consistent with the suggestion of Rakic (2009) that a larger pool of progenitors, due to an increased number of cycles of progenitor symmetric division at early embryonic stages, accounts for the difference (see Picco et al. 2018). Thus, small variations in the regulation of gene expression leading to extended prenatal growth may contribute to the large size of the human brain within the genetic envelope of the primate brain.

The increase appears particularly pronounced for the cerebral cortical gray and white matter, where it results from an expansion of the number of cortical columns (Rakic, 2009). This observation and the long-standing notion that the human cerebral cortex is of essence for advanced cognitive functions have biased attention toward the cortex, even though other brain structures, in particular the cerebellum, may have a more distinctive, human-specific molecular signature in terms of protein expression (Dumas et al. 2019; Sjöstedt et al. 2020). It can be debated, moreover, if the human neocortex is exceptionally large relative to other brain structures, considering general primate relationships (Herculano-Houzel 2009). Therefore, it may be the large absolute size, rather than relative neocortical expansion that is a hallmark of our species (Miller et al. 2019). Functional consequences of this increase range from the expansion of a long-term memory “lexicon” based on cellular neuronal engrams (Xie et al. 2014) to cognition, social behavior, and group size (Dunbar 1993).

### Increased Number of Cortical Areas

In the course of mammalian evolution from tree shrews to humans, increases in brain size and the number of neurons were accompanied by advances of the number of macroscopic brain regions. For instance, while the surface area of the cortex increased from ~105 cm^2^ per hemisphere in the macaque (Collins et al. 2010; Van Essen et al. 2012) to ~973 ± 88 cm^2^ per hemisphere in humans, the number of areas also increased, on a related, albeit slower scale. Specifically, a recent macaque parcellation included 129 areas (Van Essen et al. 2012), while in humans, an objective semiautomated neuroanatomical approach delineated more than 180 areas per hemisphere bounded by distinct changes in cortical architecture, function, connectivity, or topography (Glasser et al. 2016). This slower increase in the number of areas agrees with theoretical predictions that the number of cortical modules should scale with the root of the number of neurons, due to constraints of efficient global wiring (Braitenberg 2001).

While it is presently not fully clear which detailed molecular mechanisms led to the increase in the number of cortical areas, candidate mechanisms include gene duplication and global interactions of the developing cortical connectivity with the cortical protomap, leading to a parcellation of the human cortical ontogenetic units into a larger number of cortical areas (Rakic 1988; Cadwell et al. 2019). The increased neuron number of the human brain is also manifested by increased cortical differentiation through the addition of more elaborate, mainly superficial laminar compartments to many parts of the cerebral cortex (Barbas and García-Cabezas 2016; cf. Structural and Functional Diversity of Human Cortical Areas). Thus, the human cortex possesses a large number of cytoarchitectonically diverse compartments, as well as hierarchically structured subcompartments (Kaas 1989). As these architectonically diverse units have individual connectional fingerprints (Passingham et al. 2002), they also possess specialized functions.

The systematic increase of the number of cortical areas occurs throughout the mammalian lineage and has direct implications for the representational and memory storage capacity of the human brain, as more elements are able to accommodate a larger number and wider range of patterns corresponding to external and internal signals. Indeed, computational studies demonstrate that larger and more diverse sets of patterns can be stored and retrieved in larger recurrent neural networks, such as Hopfield networks, with low error rates (Folli et al. 2017). In line with these computational advantages is the behavioral finding that the number of neurons in the cortex of different species correlates with their cognitive performance (Herculano-Houzel 2017).

The storage capacity, particularly for long-term memories, is further increased due to the nonlinear, exponential increase in the number of potential interactions that can be made between neural elements, reflected by the additional synaptic connections that can be formed with little additional wiring cost between nearby neurons (“potential connectivity,” Chklovskii et al. 2004). This increased storage capacity appears particularly relevant for the expansion of a linguistic lexicon based on long-term cortical engrams. Interestingly, the structural storage capacity of neurons in different cortical areas based on rewiring, as indicated by the “filling fraction,” that is, the relative number of dendritic spines that could potentially be linked to nearby axonic terminations, increases along a cortical gradient from posterior (sensory) to anterior (association) areas (Stepanyants et al. 2002; Elston 2003), that is, areas that are particularly expanded in humans relative to other mammals (Fishbein et al. 2020). The observation may hint on an increased association capacity particularly of human prefrontal areas.

### Structural and Functional Diversity of Human Cortical Areas

In line with the relative uniformity of mammalian genomes, it appears that cell types are largely preserved across mammalian cortices specially at the transcriptional level (Hodge et al. 2019). Therefore, the characteristic architecture of the human brain would be expected to arise mostly from specific patterns of laminar distribution, differential protein expression, and morphological variations of the same mammalian cell types, that is, macroscopic features of human cortical architecture and connectivity, rather than actual cellular type differences. Indeed, human cortical regions show an increased differentiation, in terms of the apparent morphology and number of cortical layers. By contrast, laminar cortical structure in other mammalian species, such as for example the mouse, is much less differentiated (Charvet et al. 2014), and as a consequence, rodent cortical areas are not very clearly distinguishable from each other according to their cytoarchitecture. In a similar vein, in mice and marmosets, spine density varies only slightly across the cortex (Ballesteros-Yanez et al. 2010). By contrast, in macaque and humans, pronounced changes of spine density of pyramidal cells are observed (Elston 2003). As variations of cortical architecture are associated with variations of the intrinsic circuitry (Beul and Hilgetag 2015) as well as extrinsic connections of areas (García-Cabezas et al. 2019; Hilgetag et al. 2019), the advanced cytoarchitectonic differentiation and diversity of human cortical areas also hint on an increased diversity of intrinsic circuits and possible neural computations (Wang et al. 2019), in particular of regions involved in language processing (Galuske et al. 2000). Thus, the increased diversity of cortical cytoarchitecture and connectivity may directly contribute to the diversification of human-specific cognitive functions.

Furthermore, despite great similarities in areal organization of cortex between human and rhesus monkey, the ratio between various neuron classes included, the morphology of individual neurons in monkeys is more similar to rodents (Mohan et al. 2015). This can be supported by comparing cellular architecture, showing a decrease in neuron density and increase in neuropil volume, within analogous areas of prefrontal cortex between the rat, macaque monkey, and human, where several fold changes are seen between human and monkey, but only small differences between the rat and macaques (Džaja et al. 2019). On the other hand, in humans, there is not a disproportionately large increase in the relative size of the frontal cortices in comparison with cortex of the great apes, despite a selective increase in certain cytoarchitectonically defined areas, (such as area 10 in the prefrontal cortex) (Semendeferi et al. 2001). Thus, humans and great apes share a large frontal cortex (Semendeferi et al. 2002).

This architectonic diversity is associated with further structural and functional specializations including the biochemistry, diversity, and distribution of neurotransmitter receptors in primary sensory, motor, or multimodal association cortices (Zilles 2002; Zilles and Palomero-Gallagher 2017). This diversity is of particular interest in the case of areas involved in language processing, aware of the fact that multiple (more than 10) brain areas may contribute to language processing in adults, including the left frontal lobe, left temporal/parietal lobes, right temporal lobe, cerebellum hippocampus basal ganglia (Fedorenko et al. 2011; Deniz Can et al. 2013), and especially the prefrontal cortex (Vyshedskiy 2019). Differences in microarchitecture have been identified between Broca’s region in the human brain and areas 44 and 45 as homologs of Broca’s region in ape and macaque brains (Schenker et al. 2008, 2010; Palomero-Gallagher and Zilles 2018a) and hypothesized—together with other anatomical factors—to be responsible for the unique human ability of language. Specifically, primate interspecies differences of neuropil volume relative to cell bodies in all layers of both areas reveal an increase of neuropil volume from macaque to great apes to *H. sapiens* (Schomers et al. 2017; Palomero-Gallagher and Zilles 2018a, 2018b).

The quantitative enlargement of neuropil provides an increased opportunity for integration in local as well as long-range cortical circuitry. It may particularly facilitate the tight connectional integration of human perisylvian language areas via the arcuate fasciculus, which was shown to be an essential ingredient of the emergence of verbal working memory in recent computational studies (Schomers et al. 2017), and would, therefore, constitute a major evolutionary difference between humans and nonhuman primates.

In sum, the substantially increased size and parcellation of the human brain, comprising more and increasingly differentiated areas along spatially pronounced cortical gradients (von Economo and Koskinas 1925), may be viewed as an extension of an already existing disposition of mammalian brain evolution and framed by the genetic envelope that establishes the “proto-organization” of the brain from primate ancestors (O’Leary and Sahara 2008; Zembrzycki et al. 2015). Little, if any, additional changes at the genomic level are required, on top of those yielding a quantitative increase of brain size. These changes—associated with the relevant connectomic self-organization processes—are contributing to the increased storage capacity of the human brain as well as the functional specialization of cortical areas, culminating in connectionally linked areas specifically supporting human-specific cognitive functions, such as language.

## Sparsity and Modularity of the Cortical Connectome

### Increased Network Sparsity and Segregation

**Figure 1:**
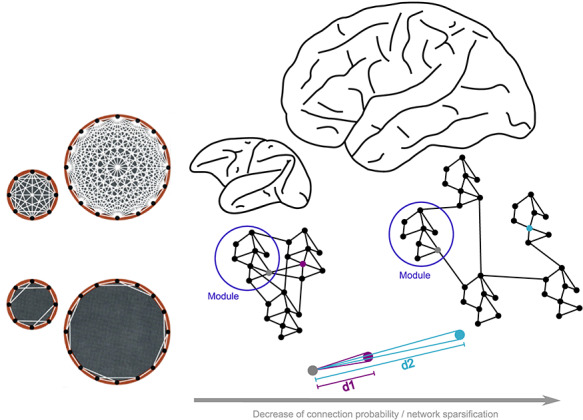
Enlargement of the brain leads to a reconfiguration of brain wiring with a relative decrease of connection density and increasing modularity. In relatively small mammalian brains, such as that of the macaque monkey, connectivity between neurons or brain areas is denser than in larger brains, such as the human brain. This is due to the fact that the average number of synaptic connections per neurons stays largely constant across mammalian brains (left bottom), rather than scaling up with the number of neurons in the network (left top), which would result in an impossible increase in white matter (Striedter 2005). Moreover, as shown on the right, when transitioning from smaller to larger brains, there is a shrinkage of the “horizon of connectional possibilities,” defined by the distance between neurons (*d1* and *d2*), leading to increased network sparsity due to a receding horizon of connectional opportunities between neurons, thus offering a parsimonious wiring constraint merely imposed by brain size changes. Note that the brain network is characterized by modules (blue circle), that is, sets of areas/neurons that are more connected in-between them when compared with the rest of the network.

The substantial neuronal expansion of the human brain has several consequences for the connectivity of the human cerebral cortex. At a fundamental level, comparative connectivity studies demonstrate that the synaptic connectivity of neurons does not scale in proportion to the overall number of neurons, but instead stays largely constant across brains of different sizes, due to volume limitations (Striedter 2005). Therefore, overall network density decreases in larger brains (Herculano-Houzel 2009). This means that the average cellular connectivity (the number of synapses relative to the number of neurons) in the human brain is much sparser than in smaller brains, and any 2 randomly selected neurons only have a tiny likelihood of being connected (Bourgeois 1997). As a rough estimate, with approximately 10^10^ neurons in the human brain, each with an average of 10^4^ synapses (Braitenberg and Schüz 1998), chance connectivity would be only 1 in a million. Moreover, projection lengths of neuronal projections, while growing in absolute terms, become relatively shorter in larger brains (Horvát et al. 2016). This shortening may be a direct consequence of the adjusted growth processes occurring in the expanded space of the human brain (Fig. 1). Thus, as an overall tendency compared with other brains, the human brain network is sparser and more locally connected and thus less well connected at the global scale.

While the increased sparsity of the human connectome may run counter to popular concepts of close brain integration by small worldness (Hilgetag and Goulas 2016), it has functional benefits, serving to separate and stabilize local representations of patterns and helping to create functional specialization. In line with this idea, computational models that seek to maximize the number of stored patterns (i.e., maximize the capacity of their lexicon) were found to be sparsely connected (Brunel 2016), and network sparsity was also shown to be stabilizing in learning models with intrinsic noise (Raman et al. 2019). A particular case is the relative sparsity of interhemispheric connections due to white matter volume limitations, which has resulted in the hemispheric specialization of cortical areas, particularly for language (Galuske et al. 2000). This asymmetry, which is in striking contrast to the general bilateral functional symmetry of mammalian brains, indeed increases the range and diversity of functional capacities of the human brain.

### Increased Network Modularity

The high average sparsity of large brain networks would quickly lead to the dissipation of signals if the networks were unstructured. However, network sparsity at the global level is counteracted locally by connections organizing into modules, that is, communities of nodes that have more connections within their home community than with nodes in other communities. Examples at different scales are cortical columns or the ventral and dorsal “streams” of the primate visual system (Hilgetag et al. 2000). These modules allow locally sustained activity while at the same time preventing global overexcitation of the networks, due to the low density of intermodular connections (Kaiser et al. 2007). Once the network grows large enough, this argument repeats at the next larger scale, implying a hierarchical, encapsulated (module within module) organization of the whole brain. Such network modules may be identified as local cortical circuits, which are contained within cortical columns, organized within cortical areas, which are themselves organized within larger systems, such as the entire visual or sensory–motor cortex. At each level, nodes are more densely wired within than between the modules (Sporns 2006). Although empirical data confirm this modular organization at some scales—for instance, for mesoscopic cortical connections of the human brain (Bassett et al. 2010; Meunier et al. 2010; Smith et al. 2019)—the detailed organization of brain networks across all scales is not yet experimentally accessible. However, it can be expected that the greater the expansion of the network, and with it the overall segregation of network elements, the greater the (hierarchical) modularity.

**Figure 2:**
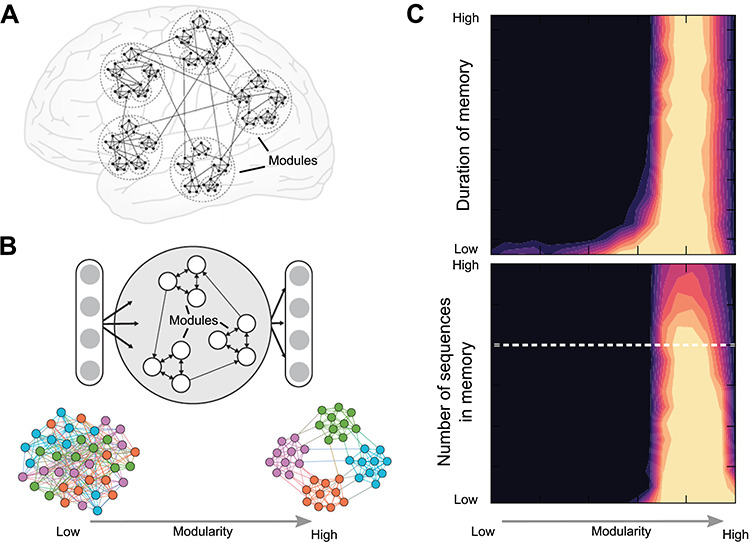
Modularity and working memory capacity. (*A*) Schematic depiction of the hierarchical modular organization of the brain’s connectome (Hilgetag and Hütt 2014). (*B*) Networks with high or low modularity forming the “reservoir” of an artificial neuronal “echo state” network (Rodriguez et al. 2019). (*C*) Functional consequences of the modular architecture of the network. The network was tested for working memory-like capacities, that is, the duration that the network could retain a sequence as well as the number of sequences that could be recalled. Note that a network configuration situated very close to high modularity exhibits the highest performance, memorizing a larger amount of sequences and retaining such memory for prolonged durations (Rodriguez et al. 2019).

Generally, modularity is a fundamental aspect of distributed yet efficiently integrated computation, balancing local integration (within the modules) with global segregation (across modules). Correspondingly, computational simulations demonstrate that modularity underlies the optimal diffusion of information across networks (Nematzadeh et al. 2014) and serves to increase the robustness of dynamic representations (Pradhan et al. 2011). Such simulations also suggest that an increase in the number of modules as well as the number of nested levels of modules serves to increase the parameter range for producing self-sustained network activity (Kaiser and Hilgetag 2010). The ability of networks to self-sustain activation patterns is a necessary precondition for the maintenance of dynamic representations underlying online short-term or working memory. Indeed, recent computational work suggests that a modular network organization, in contrast to randomly wired networks, may result in increased working memory capacity, specifically sequence memory (Rodriguez et al. 2019) (Fig. 2), with relevance for core domains of human cognition and language. Working memory temporarily stores and manages local information and can be considered as a “sketchpad of conscious thought” at the global level (Conway et al. 2003), foundational to the organization of goal-directed behavior (Miller et al. 2018; Masse et al. 2019), decision-making (Wang 2002, 2008), and conscious access (see Network Architecture and Evolution of the Global Neuronal Workspace). Interestingly, in children, working memory was found to markedly increase from 6 to about 15 years and level off between 15 and 22 years of age. Extensively trained monkeys were found less accurate than humans in working memory tasks and showed memory capacities of about 1 item (or less) against 3 in humans with the same task (Elmore et al. 2011).

Thus, modularity, conjointly with uniquely human laminar-wise connectional properties (cf. Laminar-Specific Reafference in the Human Cortex), may expand and stabilize working memory in humans—the number of items one can keep online (Goldman-Rakic 1995)—contributing to the enlargement of the linguistic lexicon and glossogeny in the context of language function. It might also underlie the ability to process unique aspects of human language, such as linear sequences of representations (stage 1 and subsequent stages of language evolution) (Fitch 2017). Moreover, the hierarchical modular organization of brain networks provides a natural topological substrate for the scaling of activity, ranging from diverse local patterns to the activation of the whole network at the global scale (Wang et al. 2011; Moretti and Muñoz 2013). The expansion of this hierarchical network organization in the expanded human brain likely increases the functional space of combining activity patterns of different lengths and sizes at different representational scales, which might underlie human cognitive abilities such as dendrophilia. In sum, as for human brain architecture, the connectomic fundamentals of the human brain can be framed by the genetic envelope that establishes the “proto-organization” of the brain from primate ancestors (O’Leary and Sahara 2008; Zembrzycki et al. 2015). Little, if any, additional changes at the genomic level are required to explain an expanded lexicon and working memory due to network sparsification and modularization, on top of those yielding a quantitative increase of brain size.

## Multilevel Processing and Global Neuronal Workspace

### Expansion of Multilevel Processing

**Figure 3:**
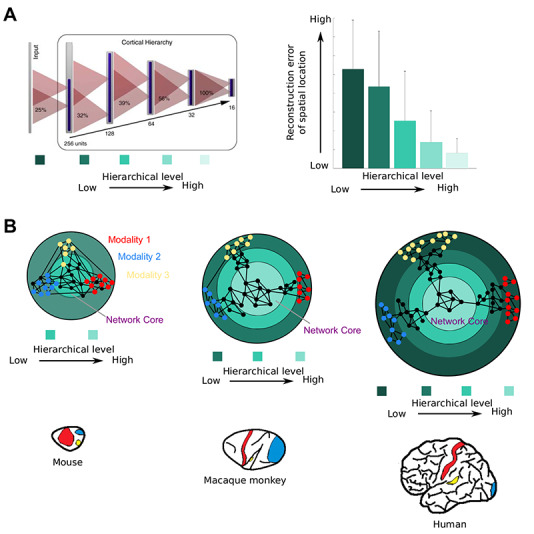
Evolution of the multilevel connectional architecture for neural representations in biological and artificial brain networks. (*A*) Multilevel artificial neural network architecture (left). A synthetic agent with a multilevel visual system can navigate a natural environment. Activity in the artificial neural network in higher levels allows a more accurate reconstruction of the location of the artificial agent (right). Note the decrease of the error of location reconstruction with increased level. Thus, a serial, convergent processing of activity from the sensorium to higher levels of the network enables abstract representations. (*B*) Enlargement of the brain and expansion of the association cortex can lead to the overall sparsification of the network (cf. Fig. 1), and, in addition, to an expanded multilevel structure of the human brain. The increased number of levels, or processing stages, defined as synaptic steps between neurons, is due to the expansion of the association cortex in humans in relation to monkeys and presumably other primates. In humans, sensory areas drift apart in physical space and, thus, do not directly connect with each other, but integrate information through a multilevel connectomic architecture toward the network core. The presence of more hierarchical levels may bestow the human brain with increased capacity for more refined and abstract representations of the sensorium. (*A*) Modified from Wyss et al. (2006). Brains in (*B*) from Krubitzer and Seelke (2012). Modality: 1 = somatosensory; 2 = auditory; 3 = visual.

As seen, the size increase of the human brain leads to increased differentiation of cortical areas that are laid out in spatially organized distributions. A further expression of multilevel processing in the brain is the convergence of signals from sensory input areas onto subsequent processing stages, which leads to increasingly larger receptive fields as well as more intricate information being represented at levels further removed from the input stage. A classic example of this convergence is the organization of the visual cortical system, where primary areas represent simple features such as oriented lines in small receptive fields, whereas subsequent areas have large receptive fields responding to complex visual features such as faces (Wagstyl et al. 2015) (Fig. 3). This kind of convergent multilevel representation is also affected by the neuronal expansion of the human brain. As there are more cortical areas in the human brain, and more differentiated areas, representations based on the interconnections of these areas also become more deeply structured and elaborate, through the interjection of further processing stages (Fig. 2, right). Studies of diverse connection architectures in the context of artificial neural networks have demonstrated that an increase in the number of intermediate layers of representation leads to more refined and accurate performance, for instance, with respect to spatial navigation (Wyss et al. 2006) (Fig. 3).

Also, at the level of the microscopic organization of the circuits, some changes in neurons ratio could produce large changes in the efficiency of neuronal processing. One of the most tremendous change is a 5-fold increase in the proportion of one GABA-neuron subclass—the calretinin neurons—in higher-order associative areas (such as the prefrontal cortex) in primates. It was proposed (Džaja et al. 2014) that an increased proportion of such neurons might allow the formation of transitory/flexible cell assemblies, which results in a supralinear increase in the number of modules in relation to the increase in the total number of principal neurons (see also Koukouli and Changeux 2020).

In addition to the neuronal organization, it was shown that the subcellular organization is also highly specialized, that is, there are nonoverlapping afferent domains of dendritic trees for neocortical excitatory connections of different sources (Petreanu et al. 2009). It is interesting to speculate about an increase in the number, or the principles of organization, of such domains through primate evolution and how this could be reflected in the efficiency of processing.

Intriguingly, such multilevel representations may preserve the original component information, if neuronal populations use coding strategies that can be mathematically described as basis functions (Deneve and Pouget 2003), and may, thus, allow simultaneous access to both the component stimuli and the combined information. An example is populations in the parietal cortex that encode visual stimuli in retinotopic coordinates modulated by body position and thereby effectively represent stimulus position relative to coordinates of the external world, rather than in body-centric reference frames (Deneve and Pouget 2003). An increase in the depth of such multilevel representations in the human brain, which follows directly from the developmental expansion particularly of the dorsolateral cortical surface, therefore, not only facilitates the creation of more intricate representations but may also lead to a highly structured “blending” of items at different levels of representation (Fauconnier and Turner 2003). This process goes beyond sensory integration and can create abstract semantic representations based on the multilevel association of multimodal sensory stimuli, as demonstrated by computational modeling (Tomasello et al. 2018). The models also show that the representations at the higher levels are less category-specific, that is, more abstract. The organization of cortical connectivity consecutive to the neuronal expansion of the human brain would, thus, be expected to increase the range and intricacy of such complex representations and more specifically the genesis of abstract and symbolic concepts. It may also favor the development of multilevel concepts, such as those required in the open-ended recursive and hierarchical organization of language (c.f. Extension of the Postnatal Development of the Human Brain and Synaptic Epigenesis).

### Network Architecture and Evolution of the Global Neuronal Workspace

**Figure 4:**
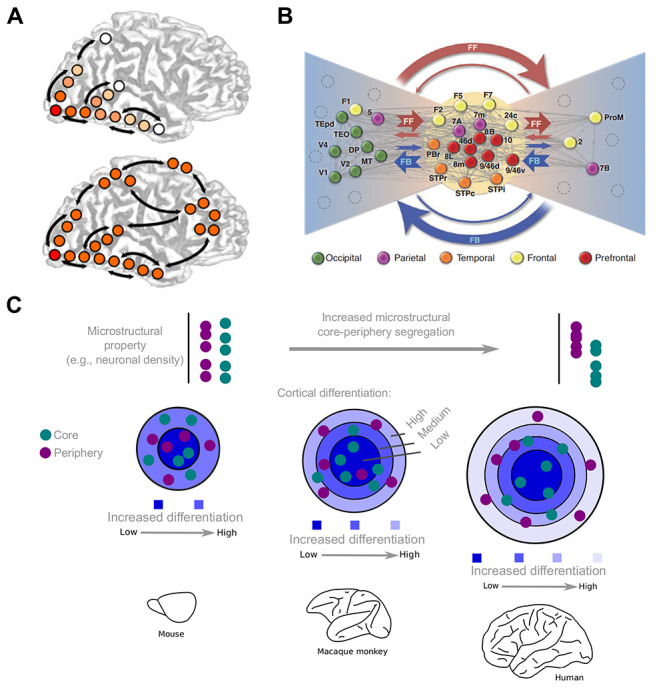
The GNW and Core-Periphery network architecture. (*A*) GNW model. The model postulates that the brain possesses a central connectional and functional component, the global workspace, composed of distributed and heavily interconnected neurons with long-range axons, in which the conscious integration of peripheral sensory input, such as visual, and emotional content takes place, giving rise to the “ignition” process (modified from Dehaene and Changeux 2011). (*B*) Network architecture of the macaque monkey cortex with a tightly interconnected and central network component (“network core”), encompassing association areas, and a less central “periphery” part of the network, encompassing mostly sensorimotor areas. Thus, the network core can be conceived as the connectomic backbone of the global workspace (adopted from Markov et al. 2013). (*C*) Situating the core–periphery network architecture within the cytoarchitectonic gradients of the cortex. A species-specific relation to the gradients of microstructural features of the cortex is observed. In progressively larger brains, core areas differ from periphery areas in terms of their cytoarchitecture, with the more topologically central core areas encompassing association areas with less laminar differentiation compared with the periphery areas, which encompass primarily sensorimotor areas with a high degree of laminar differentiation.

A characteristic level of higher cognitive functions is that of conscious processing. Several theories about the neuronal basis of conscious processing have been generated, some of which favor functional global integration (Tononi and Edelman 1998; Koch 2018), such as the integrated Information Theory (IIT) (Tononi et al. 2016), whereas others rely on specialized neuronal architectures (Adrian et al. 1954; Noebels et al. 2012) and shall be of concern here. Among them, the global neuronal workspace (GNW) hypothesis (Dehaene et al. 1998; Dehaene and Changeux 2011) offers a simple connectomic scheme based upon the contribution of neurons with long-range axons, which would form a global workspace (Baars 1988), broadcasting signals from the sensory periphery to the whole brain thus yielding “conscious” experience (Fig. 4*A*). The GNW hypothesis privileges cortical pyramidal cells with long-range excitatory axons, particularly dense in prefrontal, temporoparietal, and cingulate regions, that, together with the relevant thalamocortical loops, reciprocally interconnect multiple specialized, automatic, and nonconscious processors. In its original formulation, the GNW was designed to simulate effortful cognitive tasks and included reward mechanisms as a critical component. It was then successfully applied to fit data from simpler tasks, such as masking tasks (Dehaene and Changeux 2011; Dehaene et al. 2017). Its experimental predictions have been recently reviewed and compared with those of the IIT (Mashour et al. 2020). Its connectomic architecture has been further explored in hierarchical terms (see A Multilevel Evolution of Conscious Processing).

The GNW hypothesis relies on the presence of a reciprocally connected set of brain areas, referred to as the “neuronal workspace.” Empirical work (Goldman-Rakic 1988, 1995) has established the existence of such a highly connected set of brain areas in the mammalian connectome, also referred to as “core-periphery” or “rich-club” (Scannell and Young 1993; van den Heuvel and Sporns 2011; Ercsey-Ravasz et al. 2013; Goulas, Majka, et al. 2019b). This tightly interconnected set of areas in primates (marmoset and macaque monkeys, humans) encompasses areas of the association cortices, such as prefrontal, temporal, and parietal (Fig. 3*B*). Therefore, the core–periphery network architecture of the primate brain can be seen as the connectomic backbone of the GNW framework, with the network core corresponding to the “neuronal workspace” and the network periphery to the sensory and motor territories.

The GNW core–periphery network architecture applies to the brain networks of nonprimate mammals, including rats, mice, and cats (Scannell and Young 1993; Bota et al. 2015; Hilgetag et al. 2019), with core areas exhibiting the lowest levels of laminar differentiation and periphery areas exhibiting the highest levels of laminar differentiation (visual and somatosensory areas) (Scholtens et al. 2014; Beul et al. 2017) being species-specific (Goulas, Majka, et al. 2019b) (Fig. 3*B*, Fig. 4*C*). In mice, contrary to macaque monkeys, the network core is indistinguishable from the network periphery with respect to their degree of laminar differentiation (Goulas, Majka, et al. 2019b) (Fig. 3*C*). Since the cortical areas exhibiting the highest degree of flexibility and plasticity are the areas with low laminar differentiation (Braitenberg 1974; García-Cabezas et al. 2017), the core areas of the macaque monkey, in relation to the mouse, exhibit higher degrees of plasticity and thus may facilitate rapid learning within the primate core (Goulas, Majka, et al. 2019b). Specifically, for the case of language, a computational model implies that the regions of the core and periphery of the GNW may underlie general and category-specific meanings, respectively (Garagnani and Pulvermüller 2016).

In sum, the association of GNW, core–periphery, and cortical gradients across species indicates that the segregation of network core and periphery at the level of microstructural properties might be further pronounced in humans relative to nonhuman primates. For instance, the human core, compared with the periphery, may exhibit pronounced capacities for learning due to its microstructurally tuned composition (Fig. 4*C*). Comparative insights indicate that increasingly larger brains also entail a more pronounced segregation of core and periphery at the microstructural level (Goulas, Majka, et al. 2019b). Thus, increased brain size due to prolonged development, and thus more pronounced differences of the developmental temporal profile of brain regions, may be sufficient to result in the observed human singularities with respect to the increased microstructural segregation of core and periphery brain areas. It should be noted, however, that other gene-specific events cannot be currently excluded as factors for sculpting such configuration of the human brain.

## Laminar-Specific Reafference in the Human Cortex

### Laminar Specificity of Human Cortical Projections

**Figure 5:**
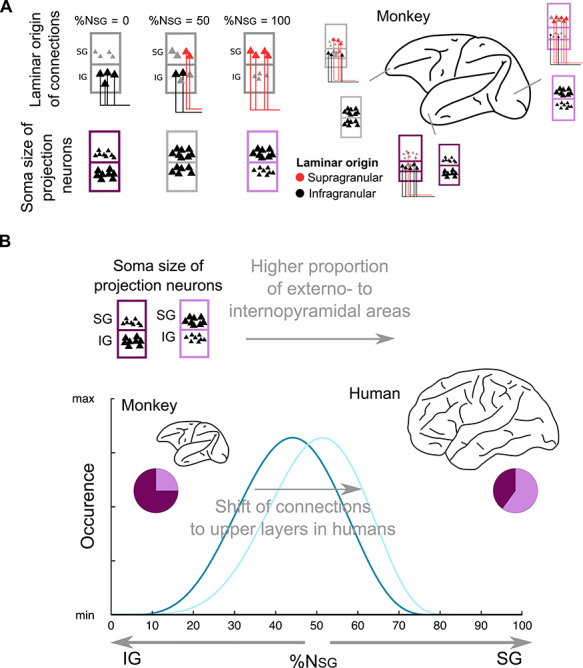
Laminar-wise “reafference shift” from monkey to human. (*A*) Laminar origin of connections is related to the cytology of the areas, specifically to soma size of the projection neurons in upper and deep layers (Goulas et al. 2018). Areas with connections emanating predominantly from deep layers (e.g., rostral temporal pole) tend to host projection neurons with larger soma size in deep compared with upper layers (interno-pyramidal areas). Areas with connections emanating predominantly from upper layers (such as peripheral visual areas) tend to host projection neurons with larger soma size in upper compared with deeper layers (externo-pyramidal areas). Areas with a more laminar-balanced origin of connections (e.g., frontal pole) also exhibit a more balanced soma size of projection neurons in upper and deeper layers (equipyramidal areas). (*B*) Qualitative observations indicate that the human cerebral cortex, relative to the monkey cortex, and presumably to other primates, exhibits a higher proportion of externo-pyramidal to interno-pyramidal areas (Sanides 1962, 1970; Sanides and Krishnamurti 1967). Due to the relation of cytology and laminar origin of connections, such cytological changes may denote a shift of the origin of long-range connections to upper layers in the human brain. Drawings modified from Goulas et al. (2018).

The cerebral cortex of mammals consists of layers that host characteristic proportions of different cell types (Brodmann 1909; von Economo and Koskinas 1925). The origins of axonal projections from different parts of the cortex (e.g., cortical areas) also exhibit layer-wise specificity (Barbas 1986; Felleman and Van Essen 1991; Goulas et al. 2018). Thus, the stratification of the cerebral cortex into layers, its laminar specificity, is a characteristic organizational feature of the mammalian cerebral cortex. With respect to axonal projections, certain areas of the cerebral cortex send axonal projections predominantly from deep layers, others predominantly from upper layers, and certain areas exhibit a balanced laminar origin of projections. In other words, the structural connections among different parts of the cerebral cortex exhibit laminar-wise specificity (Fig. 5*A*), posing the question of the characteristic human-specific organization of the laminar origin of connections.

Invasive tract-tracing studies in nonhuman animals demonstrate that the laminar origin of connections changes systematically according to the cytoarchitectonic status of cortical areas (Barbas 1986; Goulas et al. 2018; García-Cabezas et al. 2019; Hilgetag et al. 2019); specifically, areas with poor laminar differentiation, such as rostral temporal areas, predominantly send corticocortical connections from deep layers (Fig. 5*A*). Areas with pronounced laminar differentiation, such as primary visual areas, predominantly send connections from upper layers (Fig. 5*A*). Areas exhibiting intermediate laminar differentiation are characterized by a balanced to upper laminar origin of connections (Fig. 5*A*). The laminar origin of connections of each area may be related to the ratio of the soma size of projection neurons in upper versus deep layers (the phenomenon of “externo-pyramidalization”) (Goulas et al. 2018). Thus, in principle, areas that possess large projection neurons in upper (or deep) layers seem to preferentially send connections from upper (or deep) layers, while a more balanced soma size of projection neurons also entails a balanced laminar origin of connections (Fig. 5*A*).

Importantly, the relative origin of corticocortical projections, and the ratio of the soma size of projection neurons in upper versus deep layers, varies across the mammalian spectrum (Goulas et al. 2018). For example, in the mouse cortex, which shows relatively little architectonic variation across areas, corticocortical projections arise predominantly from deep cortical layers, with some involvement of upper cortical projections, but generally relatively little variation of laminar projection patterns, resulting in a “shallow cortical hierarchy” (Harris et al. 2019). This reduced spectrum of laminar corticocortical interactions associated with a less differentiated cortex was anticipated by comparative studies of mammalian cortices (Hilgetag and Grant 2010; Goulas et al. 2018; Goulas, Majka, et al. 2019b). Conversely, the architectonically highly differentiated human cortex (cf. Structural and Functional Diversity of Human Cortical Areas) is expected to be associated with a rich spectrum of laminar-specific corticocortical interaction patterns, further expanding the space of multilevel signal processing. Moreover, humans in relation to monkeys, and presumably other primates, possess more areas where large projection neurons are located in upper layers (Sanides 1962, 1970; Sanides and Krishnamurti 1967). These observations suggest that a laminar-wise reafference in the cortex, that is, a shift of the origin of connections toward upper layers, has taken place in humans (Goulas et al. 2018) (Fig. 5*B*). Single-cell transcriptomic data from mice and humans offer further empirical support for this prediction. Specifically, specific classes of projection neurons in humans that are located in upper layers (layer III) possess a transcriptome resembling that of projection neurons in deep, and not upper, layers (layers V and VI) in mice (Berg et al. 2020). In other words, homologous projection neurons in mice and humans exhibit a lower to upper layer shift in line with the aforementioned laminar-wise reafference in the human cortex (Fig. 5*B*). Importantly, this connectional shift is not necessarily tied to large brain size, since large brains of cetaceans and proboscideans do not exhibit a pronounced shift of projection neurons with large soma size in upper layers, and thus, connections in these large-cortex mammals do not show the pronounced shift of laminar origin observed in humans (Butti et al. 2011; Goulas et al. 2018).

### Shift of Laminar-Specific Reafference in Brain Hominization

If humans are characterized by a laminar-wise reafference of the cortex resulting in a shift of the origin of connections to an upper layer preference, what functional consequences can be envisioned? A series of simulations within the GNW framework, designed to represent the dynamics of masking tasks (Dehaene et al. 2003; Dehaene and Changeux 2005), were based on a multiple level architecture. Initially, a brief wave of excitation progressed through feedforward connections, then becoming amplified by its own inputs through top-down connections leading into a global self-sustained reverberating or “ignited” state. This ignition, which has been recorded in several different systems (Mashour et al. 2020), was characterized by an increased power of local corticothalamic oscillations in the gamma band and their synchrony across areas (Joglekar et al. 2018; Aru et al. 2020; Suzuki and Larkum 2020).

The GNW framework postulates that areas constituting the GNW have long-range projections that originate from upper layers (Dehaene et al. 1998). As outlined above, the preferential long-range connectivity origin from upper layers appears as a human-specific connectomic trait and cortical activity is more stable when sensory stimuli are consciously perceived (Schurger et al. 2015). Moreover, recent suggestions attribute a central role to the upper layer shift to the involvement in working memory (Joglekar et al. 2018; Miller et al. 2018). Insights from laminar-specific monkey electrophysiology indicate that gamma bursts related to the working memory delay interval are observed only in upper layers (Miller et al. 2018). Importantly, working memory is not the sole function of an individual area and its intrinsic microcircuitry (Wang et al. 2004), but a collective phenomenon of a distributed set of frontal, parietal, and temporal areas (Goldman-Rakic 1988; Christophel et al. 2017). Thus, to hold information “on-line,” gamma rhythms are important, as well as the ability to communicate with other areas within the distributed network (Rodriguez et al. 1999). In addition, “top-down” influences, mediated by slow beta frequencies (Bastos et al. 2015; Richter et al. 2018), seem to dictate which sensory information will be attended or encoded (Miller et al. 2018). More generally, gamma rhythms also appear to have a role in mediating self-control and self-awareness (Romer Thomsen et al. 2013). Thus, the shift to upper cortical projections in the human brain may have supported the enhancement of these cognitive abilities.

In sum, in humans relative to monkeys and other primates, a shift of the laminar origin of connections to upper layers might result in the human brain possessing more connections with an upper layer origin, which equips more areas with features that are considered important for working memory and conscious ignition in the GNW framework. Thus, laminar-wise reafference may lead to the emergence of an enhanced “scratchpad of conscious thought” (Miller et al. 2018) in humans. Importantly, as we describe in the next section, it is the upper layer projection neurons that exhibit prolonged postnatal maturation; thus, the contribution of upper layer connections to an enhanced conscious scratchpad can be molded by cultural and educational norms. Laminar-wise reafference is a characteristic “connectomic fundamental” of brain humanization, which is accompanied by a profound epigenetic reshuffling of the brain connectome. It does not simply derive from a scaling-up process and requires particular genetic regulatory events to occur.

## Extension of the Postnatal Development of the Human Brain and Synaptic Epigenesis

### Postnatal Extension of Human Brain Maturation

In addition to the increased prenatal development, a unique feature of the human brain is the extension of its postnatal development for up to 15 years (approximately half of the life time of *H. sapiens* at its origins) and even later, up to the third decade of life (Petanjek et al. 2011). The extension of the period of postnatal maturation results in a dramatic increase of brain volume (about a total of 5 folds) associated with a characteristic white matter expansion and enhanced neuronal connectivity (Lagercrantz 2009).

In humans, myelin develops slowly during childhood, followed by a delayed period of maturity beyond adolescence and into early adulthood. In contrast, in chimpanzees, the development of myelin already starts at a relatively more mature level at birth and ceases development long before puberty. Thus, a marked delay in the development schedule of the human neocortex plays a critical role in the growth of connections and contributes to some of our species-specific cognitive abilities (Lagercrantz 2009; Lagercrantz and Changeux 2009; Miller et al. 2012).

The “differential expression” of a few characteristic genetic regulatory events would contribute to such a quantitative increase of the postnatal developmental period of the brain, which nevertheless might already be present in the primate genetic envelope.

### Postnatal Synaptic Epigenesis of Brain Connectivity

The extended developmental period in the human species is uniquely enriched by an epigenetic self-organization of the connectivity elicited by the constant interactions of the developing infant with its physical, social, and cultural environments. Here, we use the term “epigenesis” in a sense close to its original definition by Waddington (1942) to illustrate how external events, some random, combine with inherited information coded in the genes to produce acquired connectomic variability between individuals from the same species (Changeux et al. 1973). This meaning differs from the concept of DNA “epigenetics” subsequently used in molecular biology to refer to unrelated mechanisms of DNA covalent modifications such as methylation or chromatin remodeling (Lucchesi 2018). During postnatal development, about half of the about 10^15^ adult synaptic connections are formed (at about 1 million synapses per second) and directly contribute to the formation and shaping of the synaptic architecture of the adult human brain. The development of the baby brain progresses as a multistep nested foliation resulting from successive waves of synapse outgrowth and selection (Bourgeois et al. 1986, 1994; Bourgeois and Rakic 1993). The theory, initially expressed as a mathematical model (Changeux et al. 1973), that gives access to such inscription of environmental features within the developing connectivity relies upon the variability of developing interneuronal connections and the progressive setting of robust synapses through trial-and-error mechanisms, overproduction, stabilization, and elimination processes, which formally resemble an evolutionary “Darwinian” process by variation selection (Changeux and Danchin 1976; Edelman 1978; Bourgeois et al. 1986; Kasthuri and Lichtman 2003; Bourgeois 2008; Arcaro and Livingstone 2017; Sheu et al. 2017). The model relies on the observation that at critical periods the exuberant spread and the multiple transient connectivity configurations resulting from the growth cone wanderings produce a broad diversity of synaptic connections. This diversity is then reduced, through synaptic pruning, within a given time window, by the total afferent activity, in part spontaneous but mostly originating from the reciprocal exchanges of the developing child with the outside world (Lagercrantz and Changeux 2009; Kuhl 2014; Vyshedskiy 2019). All the molecular components involved in synapse selection and stabilization are already present in the mammalian lineage and beyond (Changeux and Danchin 1976; Changeux 2017). None of them is unique to the hominization process. Another unexpected but critical feature of the theory is that it may account for the constancy of some behaviors despite high epigenetic variability of the connectivity. This idea was originally stated (Changeux et al. 1973) that “different learning inputs may produce different connective organizations and neuronal functioning abilities, but the same behavioral abilities.” Thus, the neuronal connectivity code exhibits “degeneracy” (cf. Edelman 1978; Tononi et al. 1999; Lu et al. 2009; Edelman and Gally 2013); that is, different connection patterns may carry the same input–output relationships or “meaning.”

### The Origins of the “Cultural Brain”

Evidence supporting the synapse selection model has been proposed in the case of many—vertebrate and invertebrate—developing nervous systems, in particular those developing postnatally. Among them are the visual system (Wiesel and Hubel 1963; Rakic 1976; Shatz and Stryker 1978; Le Vay et al. 1980; Blakemore 1981; Morgan et al. 2016; Arcaro et al. 2017), the neuromuscular junction (Redfern 1970; Benoit and Changeux 1978; Turney et al. 2012), the sympathetic ganglia (Lichtman and Purves 1980; Sheu et al. 2017), the cerebral cortex (Bourgeois et al. 1986, 1994; Bourgeois and Rakic 1993, 1996; Bourgeois 1997), the cerebellum (Delhaye-Bouchaud et al. 1975; Mariani and Changeux 1980), and many others (Luo and O’Leary 2005; Wu et al. 2012; Bailly et al. 2018). In humans, the overall number of synapses in the cortex peaks within the first 3 years of age then steadily declines to a plateau at around puberty (Huttenlocher and Dabholkar 1997), revealing the importance of ongoing synapse elimination, while the process of synaptic refinement goes far beyond puberty and persists in humans lifelong (Petanjek et al. 2011).

Studies in typically developing monolingual children indicate, for example, that an important period for phonetic learning occurs prior to the end of the first year. One-word utterances between the ages of 12 and 18 months, and vocabulary development “explodes” at 18 months of age (Kuhl 2011), then few words sentences (28–36 months), later inclusion of grammatical elements with “third person” reference (40–46 months) and around age 4, complete sentences of 4–5 words. On the other hand, chimpanzees never learn to combine words into a multiword “utterance” (Dehaene-Lambertz and Spelke 2015; Friederici 2020). The ability to process hierarchically structured sequences resulting in a new higher-order element—or merge/dendrophilia—has been assigned to a subpart of Broca’s area, BA44 and a fronto-temporal language network connecting in the left hemisphere language-relevant regions via dorsally located white matter fiber tracts. This dorsal fiber tract targeting Broca’s area is less developed in nonhuman primates and in prelinguistic infants than in human adults, and its development is highly correlated with the accuracy and speed with which syntactically complex sentences were understood (Friederici 2020). It is associated with the acquisition of the specific features of a given language.

Writing and reading is a recent invention which may, then, be viewed as a typical example of epigenetically led down “cultural circuits” (Changeux 1983, 2017). Historically, the first evidence for specialized writing and reading circuits in the brain was the discovery by Dejerine (1914) of pure alexia, without agraphia, resulting from circumscribed brain lesions including the supramarginal and angular gyri. New specialized sets of connections have been selected and consolidated as a consequence of written language learning, a discovery confirmed and extended by brain imaging (Castro-Caldas 1998; Carreiras et al. 2009; Dehaene et al. 2010). The connectivity used for reading and writing may, thus, be seen as an epigenetic, competitive, appropriation—rather than a “recycling” (Dehaene and Cohen 2007)—of transient brain circuits, which are selectively stabilized through teaching in the course of postnatal development around 5 years of age. These initially less specified circuits were then used, in the absence of literacy, to process alternative forms of interactions with the social environment (Goody and Watt 1963; Goody 1977; Ghirlanda et al. 2017). The example of written language illustrates how socially and culturally acquired representations might be internalized (Vygotskiĭ and Cole 1978) in the brain together with the integration of this knowledge into coherent and conscious mental syntheses (see Laminar-Specific Reafference in the Human Cortex) in the course of postnatal brain maturation (Changeux 1983; Kuhl 2011; Dehaene-Lambertz and Spelke 2015; Vyshedskiy 2019).

In connectomic terms, a nonlinear increase in the number of potential interactions takes place among the increasingly diverse microscopic and macroscopic processing units. These developments at multiple levels of “connectomic organization” expand the number and types of represented items, from sensory–motor to “symbolic” abstract representations including language with a rich lexicon, glossogeny, and teaching abilities (stages 2 and 3 of language evolution; see The Case of Language).

The synapse selection model, as mentioned, accounts for the relevant “variability” between individual brain’s connectivity and behavior, which signs their cultural belonging. This important variability would superimpose on the individual variability of the genome.

### The Developing Conscious Brain and the Origins of Language

#### A Multilevel Evolution of Conscious Processing

The evolutionary analysis together with the developmental data for the human newborn has suggested that “consciousness” is not an irreducible quality, but a bona fide brain function evolving stepwise through several nested levels of organization (Changeux 2006, 2017). At a low level “basic consciousness” would be present in the newborn infant who exhibits sensory awareness, expresses emotions, and processes mental representations (Zelazo 2004; Lagercrantz et al. 2010). At birth, all major long-distance fiber tracts are already in place (Dubois et al. 2016), although still immature. An electrophysiological signature of conscious processing—homologous to GNW ignition in adult humans—was recorded in 5-, 12-, and 15-month-old babies (Kouider et al. 2013; Dehaene-Lambertz and Spelke 2015). Explicit “self-consciousness” develops in infants at the end of the second year, together with working and episodic memory and some basic aspects of language (Posner 2007; Lou et al. 2017). This development would plausibly coincide with stage 2 and possibly 3 of language evolution (Uniformity of Mammalian Genomes).

#### Prolonged Postnatal Development of Projection Neurons: A Plausible Origin of the Theory of Mind and Language Recursivity

**Figure 6:**
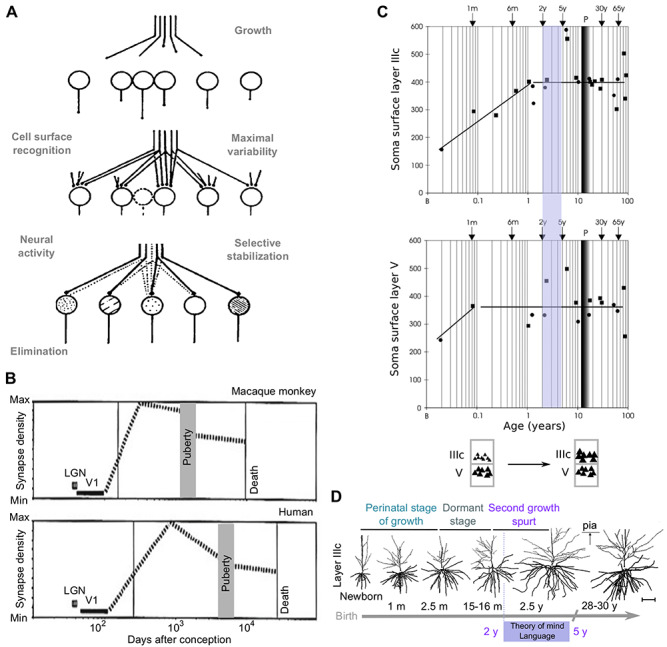
Development of dendrites and soma size of layer IIIc projection neurons and its plausible contribution to higher cognitive functions such as theory of mind and dendrophilia including language recursivity. (*A*) Model of epigenesis by selective stabilization of synapses. A nesting of many such elementary steps occurs in the course of development resulting in a hierarchical foliation of the growing networks. For a given set of developing neurons (e.g., thalamocortical or neuromuscular junction), the growing axon terminals branch exuberantly at first. But then, depending on the state of activity of the target neuron—both intrinsic spontaneous firing and evoked by external inputs—some synapses are eliminated (pruned), while others are strengthened and stabilized. In postnatal life, an important part of the activity in the network results from inputs from the environment and so the epigenetic selection of synapses represents an internalization of the outside world. (*B*) Total synapse density during the development of the monkey and human brain cortex (region V1). Note the extended time window in humans where multiple waves of synapse selection take place. Also note the sharper decrease of total synaptic density in humans before puberty, reflecting the more prominent elimination than formation of synapses. (*C*) Development of soma size of projection neurons in upper (layer IIIc) and deep layers (layer V) of the prefrontal cortex (Petanjek et al. 2008, 2019). Note that the soma size of layer IIIc neurons increases rapidly and matches or exceeds the soma size of layer V. (*D*) Dendrites of layer IIIc projection neurons have 2 phases of development. The first phase occurs perinatally, during approximately the initial 2.5 months. This initial phase is succeeded by a dormant phase. However, after the dormant phase, a second growth spurt takes place approximately at 2.5 years. Importantly, the second growth spurt characterizes upper layer (layer IIIc) projection neurons and not deep layer (layer V) projection neurons (Petanjek et al. 2008, 2019). This is approximately the age where cognitive skills like theory of mind and language recursivity start to develop (approximate span: 2–5 years), and thus, the described developmental epigenetic processes might contribute to the neurobiological basis of the “cultural brain.” (*A*) Adapted from Changeux et al. (1973). (*B*) Adapted from Bourgeois (1997). (*C*) and (*D*) adapted from Petanjek et al. (2019).

Last, the capacity to attribute mental states to other human individuals referred to as the “theory-of-mind,” which reaches full development around 3–5 years in children (Petanjek et al. 2008, 2019). A rudimentary form of “theory-of-mind” can already be seen in children around age of 2.5 years (using a simplified Sally-Anne test), whereas more mature children successfully pass a classical form of the test around age 4 (Setoh et al. 2016). Intriguingly, around 2 years, characteristic changes in the postnatal maturation of pyramidal projection neurons from the prefrontal cortex take place (Petanjek et al. 2019), which originate from upper layers—specifically layer IIIc—in the human prefrontal cortex (Goulas et al. 2018; Vyshedskiy 2019; see Shift of Laminar-Specific Reafference in Brain Hominization). These layer IIIc neurons, in contrast with deep layer V projection neurons, reach maturity between the first and third postnatal months. Between 16 months and 2.5 years, they further exhibit a unique differential increase in the number of segments and length of their basal dendrites (Fig. 6). Furthermore, a differential epigenetic elimination (pruning) of supernumerary dendritic spines has been found most pronounced and protracted on the layer IIIc neurons (Petanjek et al. 2011), especially in the prefrontal cortex (ref in Petanjek et al., 2019), a pattern also observed on oblique dendrites (Sedmak et al. 2018). Moreover, the local axonal collaterals of layer IIIc are in control of the prefrontal corticocortical output, while their long projections modulate interareal processing. They are the major integrative element of cortical processing and regulate global cortical—GNW—functioning. Thus, one may speculate that cognitive abilities, like theory of mind, at least partially, depend on the fine tuning of the still labile and adaptable long-range connections emanating from upper layer projection neurons of the human cortex that exhibit protracted maturation.

Our proposal is that this might equally be true for a unique recursive, self-embedded organization of language, including syntax (Chomsky 1957), or “merge” (Chomsky 2017) also referred to as “dendrophilia” (Fitch 2017), and/or semantic conceptual blending (Fauconnier and Turner 2003) (see The Case of Language). Without “dendrophilia,” the ability for recursive thought (thinking about one’s own thoughts) our open-ended ability to map novel thoughts onto understandable signals would be impossible.

Children automatically develop syntactic rules without explicit instruction (see The Origins of the “Cultural Brain”; Brown 1970; Marcus 1999; Dehaene-Lambertz and Spelke 2015; Friederici 2020). Between ages 2 and 4, children have concomitantly developed both syntax and theory of mind, the capacity of recursive thought together with the last stage of language evolution (Fitch 2017). We, thus, wish to propose the hypothesis that merge/dendrophilia (Dehaene et al. 2015; Berwick and Chomsky 2016, 2019; Fitch 2017; Friederici 2020; Novack and Waxman 2020) is directly related to the emergence of a new higher level of the GNW organization in late postnatal development. This new level of conscious processing would mobilize the protracted dendritic maturation of layer IIIc pyramidal neurons from prefrontal cortex, which together with their axonal collaterals and projections control prefrontal corticocortical output, as interareal processing. They become the major integrative element of cortical processing and regulate global cortical—GNW—functioning across its several levels. This mental-tree reading ability would mobilize in a concomitant top-down and bottom-up manner the multiple levels of GNW organization from the prefrontal cortex down to lower level cortical areas. Thus, human-specific cognitive abilities like theory of mind and language recursivity would at least partially depend on the fine tuning of the still labile and adaptable long-range connections emanating from upper layer projection neurons that exhibit protracted maturation. Thanks to such vertical and horizontal interconnectivity, a “global cortical synthesis” would take place at the origin of language and higher functions unique to the human brain. “Particular genetic regulatory events” may have taken place in the course of hominization to account for this important connectomic fundamental, and it has been suggested that alterations of this process might contribute to the autistic ASD phenotype in predisposed children (Petanjek et al. 2019).

Following the development of oral language around 2 to 4 years of age, additional steps of connectomic evolution that take place until the adult stage include reductions of gray matter, increases in the myelination of corticocortical connections, and changes in the architecture of large-scale cortical networks together with a reduction of the gyrification index (Klein et al. 2014). These changes concern precentral, temporal, and frontal regions, highlighting the ongoing anatomical modification of the GNW during adolescence. Furthermore, it has been suggested that schizophrenia is associated with impaired parameters of synchronous oscillations that undergo changes during the transition from adolescence to adulthood (Uhlhaas and Singer 2011).

## Conclusion

The present connectomic hypothesis provides an advanced understanding of the hominization of the brain, which plausibly accounts for several astonishing aspects of the sharp enhancement of its cognitive dispositions, including the acquisition of language, which occurred in roughly the past 2 million years, with minimal changes of genomic organization. It is still, at this stage, a working hypothesis that needs further evaluation, aware of the challenge that it lies at the convergence of functional neuroanatomy, computational modeling, and studies of higher brain function such as language. The hypothesis relies on the substantially expanded development and consequently increased size of the human brain, which may account for a number of architectonic, connectomic, and functional changes. Interestingly, the major mechanisms hypothesized to increase the efficiency of human brain connectomics are changes of specific microcircuits, which represent just a fraction of the whole network already present in nonhuman primates. In addition, the hypothesis proposes unique connectomic features, which, in synergy with the features resulting from scaled brain size, yield the “connectomic uniqueness” of the human brain and enable expanded interactions with the outside world. These features may be summarized as follows.

The remarkable size expansion of the human brain, especially of the cerebral cortex, is accompanied by an increase of the number of neurons, of cortical areas, and their architectonic differentiation together with a sparsification and increased modularity of their connectivity. These structural features support an increase of the representational capacity, in particular of the basic lexicon, and a wide diversification of the neural representations, including socio-cultural ones. Moreover, the increased modularity of connections enhances the stability of sustained activity and expands the capacity of working memory, enabling the generation of long sequences of representations and the ability to process hierarchically structured sequences. Superimposed on the increased brain size is an expanded multilevel organization of the connectome that enhances the ability for abstract processing and symbolic representations up to conscious processing. Particularly important among the singularities of human brain connectome is the shift in cortical layer reafference, which further enhances the control of working memory and the development of conscious versus nonconscious processing together with the expansion of the GNW. The human extension of pre- and postnatal postnatal development, moreover, favors an extensive increase of the epigenetic interactions of the human developing brain with its own physical, social, and cultural environments and a selection of fast-growing populations of connections under the control of its intrinsic spontaneous and environmentally evoked electrical activity. A particularly critical developmental event is, in our opinion, the supernumerary period of dendritic expansion, occurring between 2 and 4 years postnatally, associated with the development of the theory of mind and the acquisition of language recursivity or dendrophilia as plausibly manifested by the transition from *Homo heidelbergensis*/antecessor to *H. sapiens*. Along these lines, it has been noted that peak expression of synaptic genes in the prefrontal cortex is shifted from less than 1 year in chimpanzees and macaques to 5 years in humans (Liu et al. 2012).

In addition to the described postnatal connectomic development, “glossogeny” manifested by the origin, development, and internalization of culture (Vygotskiĭ and Cole 1978)**—**the “cultural brain”—develops together with the enhanced proclivity to communicate (Fishbein et al. 2020) and to epigenetically shared culturally acquired knowledge with the human-specific teaching ability or pedagogy (Premack and Premack 1996). This brain disposition, which needs to be further explored, makes possible the transgenerational transmission of knowledge and the diversification of cultures without necessary changes at the genome level, thus creating an important epigenetic interindividual variability of the brain connectome in human populations. An advanced human connectome project, therefore, needs to distinguish a “human-specific connectomic envelope” from the actual connectome of the brain of any individual human subject with its own cultural habitus (Bourdieu 1992; Finn et al. 2015).

In the course of evolution, as discussed in Gene Networks Underlying Brain Architecture and Connections (also see Boyle et al. 2017), the humanization of brain connectivity likely involved a minimal contribution of “core” genetic regulatory “events,” together with a considerable number of “peripheral” ones, which remain largely undefined at this stage despite considerable genome sequencing work (Geschwind and Rakic 2013; Somel et al. 2013; Pääbo 2014; Vallender 2014; Dumas et al. 2019; Suzuki 2020). Taking the perspective of reverse engineering, the connectomic hypothesis in contrast suggests a minimal number—6 at this stage—of “connectomic fundamentals” under the control of large scale often pleiotropic genetic regulatory events, which would quantitatively account for:

- an extended period of brain ontogenetic development,
- a consequent increase of brain size, and especially the number of cortical neurons and cortical areas,
- a scaled up multilevel organization of the connectome ultimately underlying enhanced conscious processing,
- an extended period of postnatal development with considerable epigenetic processes of synapse selection and connectomic reorganization,
- a shift of cortical layer reafference from lower to upper layers in the human cerebral cortex,
- a postnatal dendritic expansion of associative projection layer IIIc pyramidal cells, in the prefrontal cortex (while, by age 2, almost whole of dendritic growth for the vast majority of other cortical neurons had already ended), conjointly with further postnatal connectomic events, yet to be discovered.

The genetic “regulatory events,” core and peripheral, which actually determined these few “connectomic fundamentals,” and which resulted in the *H. sapiens* brain remain to be unequivocally identified. Yet, to make the proposed connectomic hypothesis empirically realistic, a few core candidate genetic regulatory events might be suggested.

Concerning the first and fourth fundamental, from an endocrine point of view, many of the genes in the ZAC1-imprinted network, including MEST, PEG3, and IGF2, are normally downregulated during postnatal development but with humans could stay active longer (Finkielstain et al. 2009). In the case of the brain protracted neuronal maturation, or neoteny, the SRGAP2C gene duplication has been mentioned (Charrier et al. 2012; Suzuki 2020). Also, transgenic rhesus monkeys carrying the human MCPH1 gene copies are claimed to show human-like neoteny of brain development (Shi et al., 2019). Many pathologies of infant brain development are associated with dysfunctions of genes functions, which might also be considered as possible candidates (van Dyck and Morrow 2017).

Regarding the second and third fundamental, the copy number of NOTCH2, SRGAP2, and ARHGAP11 genes is increased specifically in the human and exhibits pivotal functional impact on cortical development (Suzuki 2020) (see Differences in Gene Regulation) possibly together with MEF2A-mediated activity-dependent regulatory pathway (Liu et al. 2012).

As for the fifth and sixth fundamental, an enhanced regulation of NEFH, a component of neurofilaments, has been mentioned (Zeng et al. 2012; Krienen et al. 2016).

Recent formal expression of the evolutionary dynamics of the origin of language de Boer et al. (2020) has challenged the Chomskyan conjecture that language arose instantaneously in humans through a single mutation (Chomsky 1965, 2015; but Berwick and Chomsky 2016). Their analysis favors the view that “language emerged through a gradual accumulation of mutations” and also that “one needs to take into account the coevolution of genes and culture” or in our terms a few human-specific genetic “regulatory” events over the common genetic envelope of the nonhuman primates together with epigenetic imprints acquired by synaptic selection, among other postnatal events. We propose that these genetic events would predispose to the evolution of several “connectomic fundamentals.” Our hypothesis is consistent with the position of Boer et al. but may differ from it in the sense that it does not oppose the view that with the unique recursive, self-embedded organization of language that includes syntax, or “merge” (Chomsky 2017), and conceptual blending (Fauconnier and Turner 2003) appeared quite suddenly in the course of evolution as a single “syntactic event.” If according to our views the joint assembly of several connectomic fundamentals is needed simultaneously for the access to full language, the absence of any of them might prevent or switch off the merge operation connectomic phenotype. Since the development of the diverse connectomics fundamentals might, to some extent, be separately determined in the course of development, there is no reason to assume that the human-specific language phenotype arose in the course of biological evolution in a relatively short paleontological time.

Moreover, our hypothesis might be beneficial for the understanding of various psychiatric and neurological disorders where discrepancies between the level of brain anatomical perturbations and alterations of cognitive (psychomotor) abilities can be seen. One example is selective loss of large deep layer pyramids in schizophrenia and Alzheimer disease, which produces large cognitive impairment despite the absence of massive overall neuronal loss or atrophy (Hof and Morrison 2004). And vice versa, it might be assumed that sparing such neuron populations is a mechanism which would preserve cognitive functions in the cases where massive loss of other neuronal populations has taken place (Lewin 1980). The same “paradox” can be seen in some states that can be defined as atypical cognitive deficits that might help us in the understanding of the brain circuits that process high cognitive abilities (Broman and Grafman 1994). To the extent that, in such cases, despite serious intellectual impairment, some cognitive functions are well preserved and even above average (i.e., with Williams syndrome, Down syndrome, and even ASD) (Hanson et al. 2014; Bourgeron 2015; Hrvoj-Mihic and Semendeferi 2019).

Additional observations and experiments are needed to evaluate the connectomic hypothesis presented here. Among them is the exploration of connectivity features, such as sparsity, hierarchical modularity, and core–periphery segregation, which are linked together and result jointly from the evolutionary expansion of the human brain. The dynamics of such evolutionary connectomics might be implemented by computational simulations of cortical development (similar to Beul et al. 2018; Goulas, Betzel, et al. 2019a) with networks of different size and connectivity features and interaction with the sociocultural environment. The functional capabilities of these “network morphospaces” (Avena-Koenigsberger et al. 2015) might then be further examined systematically in in silico environments.

The connectomic hypothesis, thus, offers plausible answers to the interrogations, which, for different reasons, dismiss a reasonable scientific understanding of the origins of human language and of the exceptional cognitive abilities of the human brain (Mcginn 2000). On the opposite, this hypothesis gives us the opportunity to scientifically evaluate to what extent “merely quantitative differences, beyond a certain point, pass into qualitative change” (Marx 1999) in the evolutionary history of the human brain.

## Notes

We are very grateful to Jean-Pierre Bourgeois, Guillaume Dumas, Luis Quintana-Murci, Christoph Schmidt-Hieber, Paul Manger, and Mikail Rubinov for stimulating and helpful comments on this manuscript. *Conflict of Interest:* The authors declare no conflict of interest.

## Funding

Human Brain Program (SGA2 785907, SGA3 amendment 1 to J.-P.C., C.C.H); Deutsche Forschungsgemeinschaft (SPP 2041 HI 1286/7-1, SFB 936/A1, TRR 169/A2 to C.C.H.); Alexander von Humboldt Foundation (A.G.).

## References

[ref1] Adrian E, Bremer F, Jasper HH, De La Fresnaye J. 1954. Brain mechanisms and consciousness. Oxford: Blackwell Scientific Publications.

[ref2] Arcaro MJ, Livingstone MS. 2017. A hierarchical, retinotopic proto-organization of the primate visual system at birth. Elife. 6:e26196. 28671063 10.7554/eLife.26196 PMC5495573

[ref3] Arcaro MJ, Schade PF, Vincent JL, Ponce CR, Livingstone MS. 2017. Seeing faces is necessary for face-domain formation. Nat Neurosci. 20:1404–1412. 28869581 10.1038/nn.4635 PMC5679243

[ref4] Ardesch DJ, Scholtens LH, van den Heuvel MP. 2019. The human connectome from an evolutionary perspective. Prog Brain Res. 250:129–151. 31703899 10.1016/bs.pbr.2019.05.004

[ref5] Aru J, Suzuki M, Larkum ME. 2020. Cellular mechanisms of conscious processing. Trends Cogn Sci. 24:814–825. 32855048 10.1016/j.tics.2020.07.006

[ref6] Avena-Koenigsberger A, Goñi J, Solé R, Sporns O. 2015. Network morphospace. J R Soc Interface. 12:20140881. 10.1098/rsif.2014.0881 PMC4305402 25540237

[ref7] Baars BJ. 1988. A cognitive theory of consciousness. Cambridge, UK: Cambridge University Press.

[ref8] Bailly Y, Rabacchi S, Sherrard RM, Rodeau J-L, Demais V, Lohof AM, Mariani J. 2018. Elimination of all redundant climbing fiber synapses requires granule cells in the postnatal cerebellum. Sci Rep. 8:10017. 29968809 10.1038/s41598-018-28398-7 PMC6030189

[ref9] Ballesteros-Yanez I, Benavides-Piccione R, Bourgeois J-P, Changeux J-P, DeFelipe J. 2010. Alterations of cortical pyramidal neurons in mice lacking high-affinity nicotinic receptors. Proc Natl Acad Sci. 107:11567–11572. 20534523 10.1073/pnas.1006269107 PMC2895077

[ref10] Barbas H. 1986. Pattern in the laminar origin of corticocortical connections. J Comp Neurol. 252:415–422. 3793985 10.1002/cne.902520310

[ref11] Barbas H, García-Cabezas MÁ. 2016. How the prefrontal executive got its stripes. Curr Opin Neurobiol. 40:125–134. 27479655 10.1016/j.conb.2016.07.003 PMC5056826

[ref12] Bargmann CI. 2012. Beyond the connectome: how neuromodulators shape neural circuits. Bioessays. 34:458–465. 22396302 10.1002/bies.201100185

[ref13] Bassett DS, Greenfield DL, Meyer-Lindenberg A, Weinberger DR, Moore SW, Bullmore ET. 2010. Efficient physical embedding of topologically complex information processing networks in brains and computer circuits. PLoS Comput Biol. 6:e1000748. 20421990 10.1371/journal.pcbi.1000748 PMC2858671

[ref14] Bastos AM, Vezoli J, Bosman CA, Schoffelen J-M, Oostenveld R, Dowdall JR, De Weerd P, Kennedy H, Fries P. 2015. Visual areas exert feedforward and feedback influences through distinct frequency channels. Neuron. 85:390–401. 25556836 10.1016/j.neuron.2014.12.018

[ref15] Benoit P, Changeux J-P. 1978. Consequences of blocking the nerve with a local anaesthetic on the evolution of multiinnervation at the regenerating neuromuscular junction of the rat. Brain Res. 149:89–96. 656962 10.1016/0006-8993(78)90589-9

[ref16] Berg J, Sorensen SA, Ting JT, Miller JA, Chartrand T, Buchin A, Bakken TE, Budzillo A, Dee N, Ding S-L et al. 2020. Human cortical expansion involves diversification and specialization of supragranular intratelencephalic-projecting neurons. bioRxiv, 2020.2003.2031.018820.

[ref17] Berto S, Nowick K. 2018. Species-specific changes in a primate transcription factor network provide insights into the molecular evolution of the primate prefrontal cortex. Genome Biol Evol. 10:2023–2036. 30059966 10.1093/gbe/evy149 PMC6105097

[ref18] Berwick RC, Chomsky N. 2016. Why only us: language and evolution. Cambridge (MA): MIT Press.

[ref19] Berwick RC, Chomsky N. 2019. All or nothing: no half-merge and the evolution of syntax. PLoS Biol. 17:e3000539. 31774809 10.1371/journal.pbio.3000539 PMC6880979

[ref20] Beul SF, Barbas H, Hilgetag CC. 2017. A predictive structural model of the primate connectome. Sci Rep. 7:43176. 28256558 10.1038/srep43176 PMC5335700

[ref21] Beul SF, Goulas A, Hilgetag CC. 2018. Comprehensive computational modelling of the development of mammalian cortical connectivity underlying an architectonic type principle. PLoS Comput Biol. 14:e1006550. 30475798 10.1371/journal.pcbi.1006550 PMC6261046

[ref22] Beul SF, Hilgetag CC. 2015. Towards a “canonical” agranular cortical microcircuit. Front Neuroanat. 8:165. 10.3389/fnana.2014.00165 PMC4294159 25642171

[ref23] Blakemore C. 1981. Recovery from monocular deprivation in the monkey. I. Reversal of physiological effects in the visual cortex. Proc R Soc Lond B Biol Sci. 213:399–423. 6119688 10.1098/rspb.1981.0072

[ref24] Bota M, Sporns O, Swanson LW. 2015. Architecture of the cerebral cortical association connectome underlying cognition. Proc Natl Acad Sci. 112:E2093–E2101. 25848037 10.1073/pnas.1504394112 PMC4413280

[ref25] Bourdieu P. 1992. The logic of practice. Stanford, California: Stanford University Press.

[ref26] Bourgeois J. 1997. Synaptogenesis, heterochrony and epigenesis in the mammalian neocortex. Acta Paediatr. 86:27–33. 10.1111/j.1651-2227.1997.tb18340.x 9298788

[ref26a] Bourgeois J-P. 2008. Synaptogenèses normales, pathologiques et amendables dans le cortex cérébral. Psychiatr Sci Hum Neurosci. 6:124–136.

[ref27] Bourgeois J, Rakic P. 1993. Changes of synaptic density in the primary visual cortex of the macaque monkey from fetal to adult stage. J Neurosci. 13:2801–2820. 8331373 10.1523/JNEUROSCI.13-07-02801.1993 PMC6576672

[ref28] Bourgeois J-P, Goldman-Rakic PS, Rakic P. 1994. Synaptogenesis in the prefrontal cortex of rhesus monkeys. Cereb Cortex. 4:78–96. 8180493 10.1093/cercor/4.1.78

[ref29] Bourgeois J-P, Rakic P. 1996. Synaptogenesis in the occipital cortex of macaque monkey devoid of retinal input from early embryonic stages. Eur J Neurosci. 8:942–950. 8743742 10.1111/j.1460-9568.1996.tb01581.x

[ref30] Bourgeois J-P, Toutant M, Gouzé J-L, Chanceux J-P. 1986. Effect of activity on the selective stabilization of the motor innervation of fast muscle posterior latissimus dorsi from chick embryo. Int J Dev Neurosci. 4:415–429. 3455602 10.1016/0736-5748(86)90024-9

[ref31] Bourgeron T. 2015. From the genetic architecture to synaptic plasticity in autism spectrum disorder. Nat Rev Neurosci. 16:551–563. 26289574 10.1038/nrn3992

[ref32] Boyle EA, Li YI, Pritchard JK. 2017. An expanded view of complex traits: from polygenic to omnigenic. Cell. 169:1177–1186. 28622505 10.1016/j.cell.2017.05.038 PMC5536862

[ref33] Braitenberg V. 1974. Thoughts on the cerebral cortex. J Theor Biol. 46:421–447. 4420594 10.1016/0022-5193(74)90007-1

[ref34] Braitenberg V. 2001. Brain size and number of neurons: an exercise in synthetic neuroanatomy. J Comput Neurosci. 10:71–77. 11316341 10.1023/a:1008920127052

[ref35] Braitenberg V, Schüz A. 1998. Cortex: statistics and geometry of neuronal connectivity. Springer Berlin Heidelberg.

[ref36] Bräuer J, Call J, Tomasello M. 2007. Chimpanzees really know what others can see in a competitive situation. Anim Cogn. 10:439–448. 17426993 10.1007/s10071-007-0088-1

[ref37] Brodmann K. 1909. Vergleichende Lokalisationslehre der Grosshirnrinde in ihren Prinzipien dargestellt auf Grund des Zellenbaues. Leipzig: Johann Ambrosius Barth Verlag.

[ref38] Broman SH, Grafman J. 1994. Atypical cognitive deficits in developmental disorders: implications for brain function. Hillsdale, NJ: Lawrence Erlbaum Associates.

[ref39] Brown R. 1970. The first sentences of child and chimpanzee. In: Psycholinguistics: selected papers. New York: Free Press, pp. 208–231.

[ref40] Brunel N. 2016. Is cortical connectivity optimized for storing information? Nat Neurosci. 19:749–755. 27065365 10.1038/nn.4286

[ref41] Butti C, Raghanti MA, Sherwood CC, Hof PR. 2011. The neocortex of cetaceans: cytoarchitecture and comparison with other aquatic and terrestrial species: Butti et al. Ann N Y Acad Sci. 1225:47–58. 21534992 10.1111/j.1749-6632.2011.05980.x

[ref42] Cadwell CR, Bhaduri A, Mostajo-Radji MA, Keefe MG, Nowakowski TJ. 2019. Development and arealization of the cerebral cortex. Neuron. 103:980–1004. 31557462 10.1016/j.neuron.2019.07.009 PMC9245854

[ref43] Carreiras M, Seghier ML, Baquero S, Estévez A, Lozano A, Devlin JT, Price CJ. 2009. An anatomical signature for literacy. Nature. 461:983–986. 19829380 10.1038/nature08461

[ref44] Castro-Caldas A. 1998. The illiterate brain. Learning to read and write during childhood influences the functional organization of the adult brain. Brain. 121:1053–1063. 9648541 10.1093/brain/121.6.1053

[ref45] Changeux J. 1983. L’homme neuronal. Paris: Fayard.

[ref46] Changeux J-P. 2005. Allosteric mechanisms of signal transduction. Science. 308:1424–1428. 15933191 10.1126/science.1108595

[ref47] Changeux J-P. 2006. The Ferrier lecture 1998. The molecular biology of consciousness investigated with genetically modified mice. Philos Trans R Soc B Biol Sci. 361:2239–2259. 10.1098/rstb.2006.1832 PMC1764850 17015398

[ref48] Changeux J-P. 2017. Climbing brain levels of organisation from genes to consciousness. Trends Cogn Sci. 21:168–181. 28161289 10.1016/j.tics.2017.01.004

[ref49] Changeux J-P, Christopoulos A. 2017. Allosteric modulation as a unifying mechanism for receptor function and regulation. Diabetes Obes Metab. 19:4–21. 28880476 10.1111/dom.12959

[ref50] Changeux J-P, Courrege P, Danchin A. 1973. A theory of the epigenesis of neuronal networks by selective stabilization of synapses. Proc Natl Acad Sci. 70:2974–2978. 4517949 10.1073/pnas.70.10.2974 PMC427150

[ref51] Changeux JP, Danchin A. 1976. Selective stabilisation of developing synapses as a mechanism for the specification of neuronal networks. Nature. 264:705–712. 189195 10.1038/264705a0

[ref52] Charrier C, Joshi K, Coutinho-Budd J, Kim J-E, Lambert N, de Marchena J, Jin W-L, Vanderhaeghen P, Ghosh A, Sassa T et al. 2012. Inhibition of SRGAP2 function by its human-specific paralogs induces neoteny during spine maturation. Cell. 149:923–935. 22559944 10.1016/j.cell.2012.03.034 PMC3357949

[ref53] Charvet CJ, Cahalane DJ, Finlay BL. 2014. Systematic, cross-cortex variation in neuron numbers in rodents and primates. Cereb Cortex. 25:147–160. 10.1093/cercor/bht214 PMC4259279 23960207

[ref54] Cheney DL, Seyfarth RM. 1990. How monkeys see the world: inside the mind of another species. Chicago and London: University of Chicago Press.

[ref55] Chklovskii DB, Mel BW, Svoboda K. 2004. Cortical rewiring and information storage. Nature. 431:782–788. 15483599 10.1038/nature03012

[ref56] Chomsky N. 1957. Syntactic structures. Gruyter Mouton, The Hague.

[ref57] Chomsky N. 1965. Aspects of the theory of syntax. Cambridge (MA): MIT Press.

[ref58] Chomsky N. 2015. The minimalist program. 20th Anniversary Edition. Cambridge (MA); London: The MIT Press.

[ref59] Chomsky N. 2017. Language architecture and its import for evolution. Neurosci Biobehav Rev. 81:295–300. 28188889 10.1016/j.neubiorev.2017.01.053

[ref60] Christophel TB, Klink PC, Spitzer B, Roelfsema PR, Haynes J-D. 2017. The distributed nature of working memory. Trends Cogn Sci. 21:111–124. 28063661 10.1016/j.tics.2016.12.007

[ref61] Collins CE, Airey DC, Young NA, Leitch DB, Kaas JH. 2010. Neuron densities vary across and within cortical areas in primates. Proc Natl Acad Sci U S A. 107:15927–15932. 20798050 10.1073/pnas.1010356107 PMC2936588

[ref62] Conway ARA, Kane MJ, Engle RW. 2003. Working memory capacity and its relation to general intelligence. Trends Cogn Sci. 7:547–552. 14643371 10.1016/j.tics.2003.10.005

[ref63] Coupland D. 2009. Generation A. New York: Scribner.

[ref64] Darlington RB, Dunlop SA, Finlay BL. 1999. Neural development in metatherian and eutherian mammals: variation and constraint. J Comp Neurol. 411:359–368. 10413772

[ref65] de Boer B, Thompson B, Ravignani A, Boeckx C. 2020. Evolutionary dynamics do not motivate a single-mutant theory of human language. Sci Rep. 10:451. 31949223 10.1038/s41598-019-57235-8 PMC6965110

[ref66] Dediu D, Ladd DR. 2007. Linguistic tone is related to the population frequency of the adaptive haplogroups of two brain size genes, ASPM and microcephalin. Proc Natl Acad Sci. 104:10944–10949. 17537923 10.1073/pnas.0610848104 PMC1904158

[ref67] Dehaene S, Changeux J-P. 2005. Ongoing spontaneous activity controls access to consciousness: a neuronal model for inattentional blindness. PLoS Biol. 3:e141. 15819609 10.1371/journal.pbio.0030141 PMC1074751

[ref68] Dehaene S, Changeux J-P. 2011. Experimental and theoretical approaches to conscious processing. Neuron. 70:200–227. 21521609 10.1016/j.neuron.2011.03.018

[ref69] Dehaene S, Cohen L. 2007. Cultural recycling of cortical maps. Neuron. 56:384–398. 17964253 10.1016/j.neuron.2007.10.004

[ref70] Dehaene S, Kerszberg M, Changeux JP. 1998. A neuronal model of a global workspace in effortful cognitive tasks. Proc Natl Acad Sci U S A. 95:14529–14534. 9826734 10.1073/pnas.95.24.14529 PMC24407

[ref71] Dehaene S, Lau H, Kouider S. 2017. What is consciousness, and could machines have it? Science. 358:486–492. 29074769 10.1126/science.aan8871

[ref72] Dehaene S, Meyniel F, Wacongne C, Wang L, Pallier C. 2015. The neural representation of sequences: from transition probabilities to algebraic patterns and linguistic trees. Neuron. 88:2–19. 26447569 10.1016/j.neuron.2015.09.019

[ref73] Dehaene S, Pegado F, Braga LW, Ventura P, Filho GN, Jobert A, Dehaene-Lambertz G, Kolinsky R, Morais J, Cohen L. 2010. How learning to read changes the cortical networks for vision and language. Science. 330:1359–1364. 21071632 10.1126/science.1194140

[ref74] Dehaene S, Sergent C, Changeux J-P. 2003. A neuronal network model linking subjective reports and objective physiological data during conscious perception. Proc Natl Acad Sci. 100:8520–8525. 12829797 10.1073/pnas.1332574100 PMC166261

[ref75] Dehaene-Lambertz G, Spelke ES. 2015. The infancy of the human brain. Neuron. 88:93–109. 26447575 10.1016/j.neuron.2015.09.026

[ref76] Dejerine J. 1914. Semiulogie des aefectïons du système nerveux. Paris: Masson et C.

[ref77] Delhaye-Bouchaud N, Crepel F, Mariani J. 1975. Demonstration of temporary multi-innervation of the cerebellar Purkinje cells by the ascending fibers during development in the rat. C R Seances Acad Sci Ser D Sci Nat. 281:909–912. 811398

[ref78] Deneve S, Pouget A. 2003. Basis functions for object-centered representations. Neuron. 37:347–359. 12546828 10.1016/s0896-6273(02)01184-4

[ref79] Deniz Can D, Richards T, Kuhl PK. 2013. Early gray-matter and white-matter concentration in infancy predict later language skills: a whole brain voxel-based morphometry study. Brain Lang. 124:34–44. 23274797 10.1016/j.bandl.2012.10.007 PMC3551987

[ref80] Duan Z, Li F-Q, Wechsler J, Meade-White K, Williams K, Benson KF, Horwitz M. 2004. A novel notch protein, N2N, targeted by neutrophil elastase and implicated in hereditary neutropenia. Mol Cell Biol. 24:58–70. 14673143 10.1128/MCB.24.1.58-70.2004 PMC303357

[ref81] Dubois J, Adibpour P, Poupon C, Hertz-Pannier L, Dehaene-Lambertz G. 2016. MRI and M/EEG studies of the white matter development in human fetuses and infants: review and opinion. Brain Plast. 2:49–69. 29765848 10.3233/BPL-160031 PMC5928537

[ref82] Dumas G, Malesys S, Bourgeron T. 2019. Systematic detection of divergent brain proteins in human evolution and their roles in cognition (preprint). Genetics, in press. 10.1101/gr.262113.120 PMC7919455 33441416

[ref83] Dunbar RIM. 1993. Coevolution of neocortical size, group size and language in humans. Behav Brain Sci. 16:681–694.

[ref84] Džaja D, Esclapez M, Petanjek Z. 2019. Structural differences between homologous areas of prefrontal cortex of rat, monkey and human: a quantitative NeuN and calretinin immunohistochemical study. Presented at the The Brain Conferences 2019 - Dynamics of the Brain: Temporal Aspects of Computation. Rungstedgaard, Denmark.

[ref85] Džaja D, Hladnik A, Bičanić I, Baković M, Petanjek Z. 2014. Neocortical calretinin neurons in primates: increase in proportion and microcircuitry structure. Front Neuroanat. 8:103. 25309344 10.3389/fnana.2014.00103 PMC4174738

[ref86] Edelman GM. 1978. Group selection and phasic reentrant signaling: a theory of higher brain function. In: The mindful brain: cortical organization and the group-selective theory of higher brain function. Boston: MIT Press, pp. 51–98.

[ref87] Edelman GM, Gally JA. 2013. Reentry: a key mechanism for integration of brain function. Front Integr Neurosci. 7:63. 10.3389/fnint.2013.00063 PMC3753453 23986665

[ref88] Elmore LC, Ji Ma W, Magnotti JF, Leising KJ, Passaro AD, Katz JS, Wright AA. 2011. Visual short-term memory compared in rhesus monkeys and humans. Curr Biol. 21:975–979. 21596568 10.1016/j.cub.2011.04.031 PMC4634532

[ref89] Elston GN. 2003. Cortex, cognition and the cell: new insights into the pyramidal neuron and prefrontal function. Cereb Cortex. 13:1124–1138. 14576205 10.1093/cercor/bhg093

[ref90] Enard W. 2016. The molecular basis of human brain evolution. Curr Biol. 26:R1109–R1117. 27780052 10.1016/j.cub.2016.09.030

[ref91] Ercsey-Ravasz M, Markov NT, Lamy C, Van Essen DC, Knoblauch K, Toroczkai Z, Kennedy H. 2013. A predictive network model of cerebral cortical connectivity based on a distance rule. Neuron. 80:184–197. 24094111 10.1016/j.neuron.2013.07.036 PMC3954498

[ref92] Fauconnier G, Turner M. 2003. Conceptual blending, form and meaning. Rech En Commun. 19:30.

[ref93] Fedorenko E, Behr MK, Kanwisher N. 2011. Functional specificity for high-level linguistic processing in the human brain. Proc Natl Acad Sci. 108:16428–16433. 21885736 10.1073/pnas.1112937108 PMC3182706

[ref94] Felleman DJ, Van Essen DC. 1991. Distributed hierarchical processing in the primate cerebral cortex. Cereb Cortex. 1:1–47. 1822724 10.1093/cercor/1.1.1-a

[ref95] Fiddes IT, Lodewijk GA, Mooring M, Bosworth CM, Ewing AD, Mantalas GL, Novak AM, van den Bout A, Bishara A, Rosenkrantz JL et al. 2018. Human-specific NOTCH2NL genes affect notch signaling and cortical neurogenesis. Cell. 173:1356–1369.e22. 29856954 10.1016/j.cell.2018.03.051 PMC5986104

[ref96] Finkielstain GP, Forcinito P, Lui JCK, Barnes KM, Marino R, Makaroun S, Nguyen V, Lazarus JE, Nilsson O, Baron J. 2009. An extensive genetic program occurring during postnatal growth in multiple tissues. Endocrinology. 150:1791–1800. 19036884 10.1210/en.2008-0868 PMC2659288

[ref97] Finlay B, Darlington R. 1995. Linked regularities in the development and evolution of mammalian brains. Science. 268:1578–1584. 7777856 10.1126/science.7777856

[ref98] Finn ES, Shen X, Scheinost D, Rosenberg MD, Huang J, Chun MM, Papademetris X, Constable RT. 2015. Functional connectome fingerprinting: identifying individuals using patterns of brain connectivity. Nat Neurosci. 18:1664–1671. 26457551 10.1038/nn.4135 PMC5008686

[ref99] Fishbein AR, Fritz JB, Idsardi WJ, Wilkinson GS. 2020. What can animal communication teach us about human language? Philos Trans R Soc B Biol Sci. 375:20190042. 10.1098/rstb.2019.0042 PMC6895550 31735148

[ref100] Fitch WT. 2017. Empirical approaches to the study of language evolution. Psychon Bull Rev. 24:3–33. 28150125 10.3758/s13423-017-1236-5

[ref101] Fitch WT. 2020. Animal cognition and the evolution of human language: why we cannot focus solely on communication. Philos Trans R Soc B Biol Sci. 375:20190046. 10.1098/rstb.2019.0046 PMC6895558 31735146

[ref102] Florio M, Albert M, Taverna E, Namba T, Brandl H, Lewitus E, Haffner C, Sykes A, Wong FK, Peters J et al. 2015. Human-specific gene ARHGAP11B promotes basal progenitor amplification and neocortex expansion. Science. 347:1465–1470. 25721503 10.1126/science.aaa1975

[ref103] Florio M, Heide M, Pinson A, Brandl H, Albert M, Winkler S, Wimberger P, Huttner WB, Hiller M. 2018. Evolution and cell-type specificity of human-specific genes preferentially expressed in progenitors of fetal neocortex. Elife. 7:e32332. 29561261 10.7554/eLife.32332 PMC5898914

[ref104] Florio M, Namba T, Pääbo S, Hiller M, Huttner WB. 2016. A single splice site mutation in human-specific *ARHGAP11B* causes basal progenitor amplification. Sci Adv. 2:e1601941. 27957544 10.1126/sciadv.1601941 PMC5142801

[ref105] Folli V, Leonetti M, Ruocco G. 2017. On the maximum storage capacity of the Hopfield model. Front Comput Neurosci. 10:144. 10.3389/fncom.2016.00144 PMC5222833 28119595

[ref106] Fossati M, Pizzarelli R, Schmidt ER, Kupferman JV, Stroebel D, Polleux F, Charrier C. 2016. SRGAP2 and its human-specific paralog co-regulate the development of excitatory and inhibitory synapses. Neuron. 91:356–369. 27373832 10.1016/j.neuron.2016.06.013 PMC5385270

[ref107] Friederici AD. 2017. Language in our brain: the origins of a uniquely human capacity. Cambridge (MA): The MIT Press.

[ref108] Friederici AD. 2020. Hierarchy processing in human neurobiology: how specific is it? Philos Trans R Soc B Biol Sci. 375:20180391. 10.1098/rstb.2018.0391 PMC6895560 31735144

[ref109] Galuske RA, Schlote W, Bratzke H, Singer W. 2000. Interhemispheric asymmetries of the modular structure in human temporal cortex. Science. 289:1946–1949. 10988077 10.1126/science.289.5486.1946

[ref110] Garagnani M, Pulvermüller F. 2016. Conceptual grounding of language in action and perception: a neurocomputational model of the emergence of category specificity and semantic hubs. Eur J Neurosci. 43:721–737. 26660067 10.1111/ejn.13145 PMC4982106

[ref111] García-Cabezas MÁ, Joyce MKP, John YJ, Zikopoulos B, Barbas H. 2017. Mirror trends of plasticity and stability indicators in primate prefrontal cortex. Eur J Neurosci. 46:2392–2405. 28921934 10.1111/ejn.13706 PMC5656436

[ref112] García-Cabezas MÁ, Zikopoulos B, Barbas H. 2019. The structural model: a theory linking connections, plasticity, pathology, development and evolution of the cerebral cortex. Brain Struct Funct. 224:985–1008. 30739157 10.1007/s00429-019-01841-9 PMC6500485

[ref113] Geschwind DH, Rakic P. 2013. Cortical evolution: judge the brain by its cover. Neuron. 80:633–647. 24183016 10.1016/j.neuron.2013.10.045 PMC3922239

[ref114] Ghirlanda S, Lind J, Enquist M. 2017. Memory for stimulus sequences: a divide between humans and other animals? R Soc Open Sci. 4:161011. 28680660 10.1098/rsos.161011 PMC5493902

[ref115] Glasser MF, Coalson TS, Robinson EC, Hacker CD, Harwell J, Yacoub E, Ugurbil K, Andersson J, Beckmann CF, Jenkinson M et al. 2016. A multi-modal parcellation of human cerebral cortex. Nature. 536:171–178. 27437579 10.1038/nature18933 PMC4990127

[ref116] Goldman-Rakic PS. 1988. Topography of cognition: parallel distributed networks in primate association cortex. Ann Rev Neurosci. 11:137–156. 3284439 10.1146/annurev.ne.11.030188.001033

[ref117] Goldman-Rakic PS. 1995. Cellular basis of working memory. Neuron. 14:477–485. 7695894 10.1016/0896-6273(95)90304-6

[ref118] Goody J. 1977. The domestication of the savage mind. Cambridge, UK: Cambridge University Press.

[ref119] Goody J, Watt I. 1963. The consequences of literacy. Comp Stud Soc Hist. 5:304–345.

[ref120] Goulas A, Betzel RF, Hilgetag CC. 2019a. Spatiotemporal ontogeny of brain wiring. Sci Adv. 5:eaav9694. 31206020 10.1126/sciadv.aav9694 PMC6561744

[ref121] Goulas A, Majka P, Rosa MGP, Hilgetag CC. 2019b. A blueprint of mammalian cortical connectomes. PLoS Biol. 17:e2005346. 30901324 10.1371/journal.pbio.2005346 PMC6456226

[ref122] Goulas A, Zilles K, Hilgetag CC. 2018. Cortical gradients and laminar projections in mammals. Trends Neurosci. 41:775–788. 29980393 10.1016/j.tins.2018.06.003

[ref123] Greenspan RJ. 2009. Selection, gene interaction, and flexible gene networks. Cold Spring Harb Symp Quant Biol. 74:131–138. 19903749 10.1101/sqb.2009.74.029

[ref124] Hanson KL, Hrvoj-Mihic B, Semendeferi K. 2014. A dual comparative approach: integrating lines of evidence from human evolutionary neuroanatomy and neurodevelopmental disorders. Brain Behav Evol. 84:135–155. 25247986 10.1159/000365409 PMC4174449

[ref125] Harris JA, Mihalas S, Hirokawa KE, Whitesell JD, Choi H, Bernard A, Bohn P, Caldejon S, Casal L, Cho A et al. 2019. Hierarchical organization of cortical and thalamic connectivity. Nature. 575:195–202. 31666704 10.1038/s41586-019-1716-z PMC8433044

[ref126] Herculano-Houzel S. 2009. The human brain in numbers: a linearly scaled-up primate brain. Front Hum Neurosci. 3:31. 19915731 10.3389/neuro.09.031.2009 PMC2776484

[ref127] Herculano-Houzel S. 2016. The human advantage: a new understanding of how our brain became remarkable. Cambridge, MA: The MIT Press.

[ref128] Herculano-Houzel S. 2017. Numbers of neurons as biological correlates of cognitive capability. Curr Opin Behav Sci. 16:1–7.

[ref129] Hilgetag CC, Beul SF, van Albada SJ, Goulas A. 2019. An architectonic type principle integrates macroscopic cortico-cortical connections with intrinsic cortical circuits of the primate brain. Netw Neurosci. 3:905–923. 31637331 10.1162/netn_a_00100 PMC6777964

[ref130] Hilgetag CC, Burns G, O’Neill MA, Scannell JW, Young MP. 2000. Anatomical connectivity defines the organization of clusters of cortical areas in the macaque monkey and the cat. Philos Trans R Soc Lond Ser B-Biol Sci. 355:91–110. 10703046 10.1098/rstb.2000.0551 PMC1692723

[ref131] Hilgetag CC, Goulas A. 2016. Is the brain really a small-world network? Brain Struct Funct. 221:2361–2366. 25894630 10.1007/s00429-015-1035-6 PMC4853440

[ref132] Hilgetag CC, Grant S. 2010. Cytoarchitectural differences are a key determinant of laminar projection origins in the visual cortex. Neuroimage. 51:1006–1017. 20211270 10.1016/j.neuroimage.2010.03.006

[ref133] Hilgetag CC, Hütt M-T. 2014. Hierarchical modular brain connectivity is a stretch for criticality. Trends Cogn Sci. 18:114–115. 24268289 10.1016/j.tics.2013.10.016

[ref134] Hobert O, Kratsios P. 2019. Neuronal identity control by terminal selectors in worms, flies, and chordates. Curr Opin Neurobiol. 56:97–105. 30665084 10.1016/j.conb.2018.12.006

[ref135] Hodge RD, Bakken TE, Miller JA, Smith KA, Barkan ER, Graybuck LT, Close JL, Long B, Johansen N, Penn O et al. 2019. Conserved cell types with divergent features in human versus mouse cortex. Nature. 573:61–68. 31435019 10.1038/s41586-019-1506-7 PMC6919571

[ref136] Hof PR, Morrison JH. 2004. The aging brain: morphomolecular senescence of cortical circuits. Trends Neurosci. 27:607–613. 15374672 10.1016/j.tins.2004.07.013

[ref137] Holloway AK, Bruneau BG, Sukonnik T, Rubenstein JL, Pollard KS. 2016. Accelerated evolution of enhancer hotspots in the mammal ancestor. Mol Biol Evol. 33:1008–1018. 26715627 10.1093/molbev/msv344 PMC4776709

[ref138] Horvát S, Gămănu R, Ercsey-Ravasz M, Magrou L, Gămănu B, Van Essen DC, Burkhalter A, Knoblauch K, Toroczkai Z, Kennedy H. 2016. Spatial embedding and wiring cost constrain the functional layout of the cortical network of rodents and primates. PLoS Biol. 14:e1002512. 27441598 10.1371/journal.pbio.1002512 PMC4956175

[ref139] Hrvoj-Mihic B, Semendeferi K. 2019. Neurodevelopmental disorders of the prefrontal cortex in an evolutionary context. Prog Brain Res. 250:109–127. 31703898 10.1016/bs.pbr.2019.05.003

[ref140] Hsieh P, Vollger MR, Dang V, Porubsky D, Baker C, Cantsilieris S, Hoekzema K, Lewis AP, Munson KM, Sorensen M et al. 2019. Adaptive archaic introgression of copy number variants and the discovery of previously unknown human genes. Science. 366:eaax2083. 31624180 10.1126/science.aax2083 PMC6860971

[ref141] Huang AY, Li P, Rodin RE, Kim SN, Dou Y, Kenny CJ, Akula SK, Hodge RD, Bakken TE, Miller JA et al. 2020. Parallel RNA and DNA analysis after deep sequencing (PRDD-seq) reveals cell type-specific lineage patterns in human brain. Proc Natl Acad Sci. 117:13886–13895. 32522880 10.1073/pnas.2006163117 PMC7322034

[ref142] Hurford J. 1990. Nativist and functional explanations in language acquisition. In: Roca IM, editor. Logical issues in language acquisition. Dordrecht: Foris, pp. 85–136.

[ref143] Huttenlocher PR, Dabholkar AS. 1997. Regional differences in synaptogenesis in human cerebral cortex. J Comp Neurol. 387:167–178. 9336221 10.1002/(sici)1096-9861(19971020)387:2<167::aid-cne1>3.0.co;2-z

[ref144] Joglekar MR, Mejias JF, Yang GR, Wang X-J. 2018. Inter-areal balanced amplification enhances signal propagation in a large-scale circuit model of the primate cortex. Neuron. 98:222–234.e8. 29576389 10.1016/j.neuron.2018.02.031

[ref145] Jukic AM, Baird DD, Weinberg CR, McConnaughey DR, Wilcox AJ. 2013. Length of human pregnancy and contributors to its natural variation. Hum Reprod. 28:2848–2855. 23922246 10.1093/humrep/det297 PMC3777570

[ref146] Kaas J. 1989. Why does the brain have so many visual areas? J Cogn Neurosci. 1:121–135. 23968461 10.1162/jocn.1989.1.2.121

[ref147] Kaiser M, Görner M, Hilgetag C. 2007. Criticality of spreading dynamics in hierarchical cluster networks without inhibition. New J Phys. 9:110.

[ref148] Kaiser M, Hilgetag CC. 2010. Optimal hierarchical modular topologies for producing limited sustained activation of neural networks. Front Neuroinform. 4:8. 20514144 10.3389/fninf.2010.00008 PMC2876872

[ref149] Kalebic N, Gilardi C, Albert M, Namba T, Long KR, Kostic M, Langen B, Huttner WB. 2018. Human-specific ARHGAP11B induces hallmarks of neocortical expansion in developing ferret neocortex. Elife. 7:e41241. 30484771 10.7554/eLife.41241 PMC6303107

[ref150] Kaminski J. 2004. Word learning in a domestic dog: evidence for “fast mapping.”. Science. 304:1682–1683. 15192233 10.1126/science.1097859

[ref26b] Kasthuri N, Lichtman JW. 2003. The role of neuronal identity in synaptic competition. Nature. 424:426–430. 12879070 10.1038/nature01836

[ref151] Klein D, Rotarska-Jagiela A, Genc E, Sritharan S, Mohr H, Roux F, Han CE, Kaiser M, Singer W, Uhlhaas PJ. 2014. Adolescent brain maturation and cortical folding: evidence for reductions in gyrification. PLoS One. 9:e84914. 24454765 10.1371/journal.pone.0084914 PMC3893168

[ref152] Ko H, Hofer SB, Pichler B, Buchanan KA, Sjöström PJ, Mrsic-Flogel TD. 2011. Functional specificity of local synaptic connections in neocortical networks. Nature. 473:87–91. 21478872 10.1038/nature09880 PMC3089591

[ref153] Koch C. 2018. What is consciousness? Nature. 557:S8–S12. 29743705 10.1038/d41586-018-05097-x

[ref154] Kouider S, Stahlhut C, Gelskov SV, Barbosa LS, Dutat M, de Gardelle V, Christophe A, Dehaene S, Dehaene-Lambertz G. 2013. A neural marker of perceptual consciousness in infants. Science. 340:376–380. 23599498 10.1126/science.1232509

[ref155] Koukouli F, Changeux J-P. 2020. Do nicotinic receptors modulate high-order cognitive processing? Trends Neurosci. 43:550–564. 32591156 10.1016/j.tins.2020.06.001

[ref156] Krienen FM, Yeo BTT, Ge T, Buckner RL, Sherwood CC. 2016. Transcriptional profiles of supragranular-enriched genes associate with corticocortical network architecture in the human brain. Proc Natl Acad Sci. 113:E469–E478. 26739559 10.1073/pnas.1510903113 PMC4739529

[ref157] Krubitzer LA, Seelke AMH. 2012. Cortical evolution in mammals: the bane and beauty of phenotypic variability. Proc Natl Acad Sci. 109:10647–10654. 22723368 10.1073/pnas.1201891109 PMC3386882

[ref158] Kuhl PK. 2000. A new view of language acquisition. Proc Natl Acad Sci. 97:11850–11857. 11050219 10.1073/pnas.97.22.11850 PMC34178

[ref159] Kuhl PK. 2011. Early language learning and literacy: neuroscience implications for education. Mind Brain Educ. 5:128–142. 21892359 10.1111/j.1751-228X.2011.01121.x PMC3164118

[ref160] Kuhl PK. 2014. Early language learning and the social brain. Cold Spring Harb Symp Quant Biol. 79:211–220. 25943768 10.1101/sqb.2014.79.024802

[ref161] Lagercrantz H. 2009. The birth of consciousness. Early Hum Dev. 85:S57–S58. 19762170 10.1016/j.earlhumdev.2009.08.017

[ref162] Lagercrantz H, Changeux J-P. 2009. The emergence of human consciousness: from fetal to neonatal life. Pediatr Res. 65:255–260. 19092726 10.1203/PDR.0b013e3181973b0d

[ref163] Lagercrantz H, Hanson MA, Ment LR, Peebles DM. 2010. The newborn brain: neuroscience and clinical applications. 2nd ed. Cambridge, UK: Cambridge University Press.

[ref164] Laland KN. 2017. The origins of language in teaching. Psychon Bull Rev. 24:225–231. 27368625 10.3758/s13423-016-1077-7 PMC5325857

[ref165] Le Vay S, Wiesel TN, Hubel DH. 1980. The development of ocular dominance columns in normal and visually deprived monkeys. J Comp Neurol. 191:1–51. 6772696 10.1002/cne.901910102

[ref166] Lewin R. 1980. Is your brain really necessary? Science. 210:1232–1234. 7434023 10.1126/science.7434023

[ref167] Lichtman JW, Purves D. 1980. The elimination of redundant preganglionic innervation to hamster sympathetic ganglion cells in early post-natal life. J Physiol. 301:213–228. 7411428 10.1113/jphysiol.1980.sp013200 PMC1279393

[ref168] Liu X, Somel M, Tang L, Yan Z, Jiang X, Guo S, Yuan Y, He L, Oleksiak A, Zhang Y et al. 2012. Extension of cortical synaptic development distinguishes humans from chimpanzees and macaques. Genome Res. 22:611–622. 22300767 10.1101/gr.127324.111 PMC3317144

[ref169] Loh P-R, Bhatia G, Gusev A, Finucane HK, Bulik-Sullivan BK, Pollack SJ, Teresa R de Candia, Schizophrenia Working Group of the Psychiatric Genomics Consortium, Lee SH, Wray NR et al. 2015. Contrasting genetic architectures of schizophrenia and other complex diseases using fast variance-components analysis. Nat Genet. 47:1385–1392. 26523775 10.1038/ng.3431 PMC4666835

[ref170] Lou HC, Changeux JP, Rosenstand A. 2017. Towards a cognitive neuroscience of self-awareness. Neurosci Biobehav Rev. 83:765–773. 27079562 10.1016/j.neubiorev.2016.04.004

[ref171] Lu J, Tapia J, White O, Lichtman J. 2009. The Interscutularis muscle Connectome. PLoS Biol. 7:e32. 19209956 10.1371/journal.pbio.1000032 PMC2637925

[ref172] Lucchesi JC. 2018. Transcriptional modulation of entire chromosomes: dosage compensation. J Genet. 97:357–364. 29932054

[ref173] Luo L, O’Leary DDM. 2005. Axon retraction and degeneration in development and disease. Annu Rev Neurosci. 28:127–156. 16022592 10.1146/annurev.neuro.28.061604.135632

[ref174] Marchetto MC, Hrvoj-Mihic B, Kerman BE, Yu DX, Vadodaria KC, Linker SB, Narvaiza I, Santos R, Denli AM, Mendes AP et al. 2019. Species-specific maturation profiles of human, chimpanzee and bonobo neural cells. Elife. 8:e37527. 30730291 10.7554/eLife.37527 PMC6366899

[ref175] Marcus GF. 1999. Rule learning by seven-month-old infants. Science. 283:77–80. 9872745 10.1126/science.283.5398.77

[ref176] Mariani J, Changeux J-P. 1980. Intracellular recordings of the multiple innervation of Purkinje cells by climbing fibers in the cerebellum of the developing rat. C R Seances Acad Sci Ser D Sci Nat. 29:97–100. 6774831

[ref177] Markov NT, Ercsey-Ravasz M, Van Essen DC, Knoblauch K, Toroczkai Z, Kennedy H. 2013. Cortical high-density counterstream architectures. Science. 342:1238406. 24179228 10.1126/science.1238406 PMC3905047

[ref178] Marx K. 1999. Capital: an abridged edition. Oxford: OUP.

[ref179] Mashour GA, Roelfsema P, Changeux J-P, Dehaene S. 2020. Conscious processing and the global neuronal workspace hypothesis. Neuron. 105:776–798. 32135090 10.1016/j.neuron.2020.01.026 PMC8770991

[ref180] Masse NY, Yang GR, Song HF, Wang X-J, Freedman DJ. 2019. Circuit mechanisms for the maintenance and manipulation of information in working memory. Nat Neurosci. 22:1159–1167. 31182866 10.1038/s41593-019-0414-3 PMC7321806

[ref181] Mcginn C. 2000. The mysterious flame- conscious minds in a material world. New York, NY.

[ref182] McLean CY, Reno PL, Pollen AA, Bassan AI, Capellini TD, Guenther C, Indjeian VB, Lim X, Menke DB, Schaar BT et al. 2011. Human-specific loss of regulatory DNA and the evolution of human-specific traits. Nature. 471:216–219. 21390129 10.1038/nature09774 PMC3071156

[ref183] Meunier D, Lambiotte R, Bullmore ET. 2010. Modular and hierarchically modular organization of brain networks. Front Neurosci. 4:200. 21151783 10.3389/fnins.2010.00200 PMC3000003

[ref184] Miller DJ, Duka T, Stimpson CD, Schapiro SJ, Baze WB, McArthur MJ, Fobbs AJ, Sousa AMM, Sestan N, Wildman DE et al. 2012. Prolonged myelination in human neocortical evolution. Proc Natl Acad Sci. 109:16480–16485. 23012402 10.1073/pnas.1117943109 PMC3478650

[ref185] Miller EK, Lundqvist M, Bastos AM. 2018. Working memory 2.0. Neuron. 100:463–475. 30359609 10.1016/j.neuron.2018.09.023 PMC8112390

[ref186] Miller IF, Barton RA, Nunn CL. 2019. Quantitative uniqueness of human brain evolution revealed through phylogenetic comparative analysis. Elife. 8:e41250. 10.7554/eLife.41250 PMC6379089 30702428

[ref187] Mohan H, Verhoog MB, Doreswamy KK, Eyal G, Aardse R, Lodder BN, Goriounova NA, Asamoah B. 2015. Dendritic and axonal architecture of individual pyramidal neurons across layers of adult human neocortex. Cereb Cortex. 25:4839–4853. 26318661 10.1093/cercor/bhv188 PMC4635923

[ref188] Mora-Bermúdez F, Badsha F, Kanton S, Camp JG, Vernot B, Köhler K, Voigt B, Okita K, Maricic T, He Z et al. 2016. Differences and similarities between human and chimpanzee neural progenitors during cerebral cortex development. Elife. 5:e18683. 10.7554/eLife.18683 PMC5110243 27669147

[ref189] Moretti P, Muñoz MA. 2013. Griffiths phases and the stretching of criticality in brain networks. Nat Commun. 4:2521. 24088740 10.1038/ncomms3521

[ref190] Morgan JL, Berger DR, Wetzel AW, Lichtman JW. 2016. The fuzzy logic of network connectivity in mouse visual thalamus. Cell. 165:192–206. 27015312 10.1016/j.cell.2016.02.033 PMC4808248

[ref191] Mozzi A, Guerini FR, Forni D, Costa AS, Nemni R, Baglio F, Cabinio M, Riva S, Pontremoli C, Clerici M et al. 2017. REST, a master regulator of neurogenesis, evolved under strong positive selection in humans and in nonhuman primates. Sci Rep. 7:9530. 28842657 10.1038/s41598-017-10245-w PMC5573535

[ref192] Nematzadeh A, Ferrara E, Flammini A, Ahn Y-Y. 2014. Optimal network modularity for information diffusion. Phys Rev Lett. 113:088701. 25192129 10.1103/PhysRevLett.113.088701

[ref193] Noebels JL, Avoli M, Rogawski MA, Olsen RW, Delgado-Escueta AV, editors. 2012. Jasper’s basic mechanisms of the epilepsies [Internet]. 4th edition Bethesda (MD): National Center for Biotechnology Information (US). doi: 10.1093/med/9780199746545.001.0001. 22787592

[ref194] Northcutt GR, Kaas JH. 1995. The emergence and evolution of mammalian neocortex. Trends Neurosci. 18:373–379. 7482801 10.1016/0166-2236(95)93932-n

[ref195] Novack MA, Waxman S. 2020. Becoming human: human infants link language and cognition, but what about the other great apes? Philos Trans R Soc B Biol Sci. 375:20180408. 10.1098/rstb.2018.0408 PMC6895556 31735145

[ref196] Ohno S. 1999. Gene duplication and the uniqueness of vertebrate genomes circa 1970–1999. Semin Cell Dev Biol. 10:517–522. 10597635 10.1006/scdb.1999.0332

[ref197] O’Leary DD, Sahara S. 2008. Genetic regulation of arealization of the neocortex. Curr Opin Neurobiol. 18:90–100. 18524571 10.1016/j.conb.2008.05.011 PMC2677555

[ref198] Pääbo S. 2014. The human condition—a molecular approach. Cell. 157:216–226. 24679537 10.1016/j.cell.2013.12.036

[ref199] Palomero-Gallagher N, Zilles K. 2018a. Differences in cytoarchitecture of Broca’s region between human, ape and macaque brains. Cortex. 118:132–153. 30333085 10.1016/j.cortex.2018.09.008

[ref200] Palomero-Gallagher N, Zilles K. 2018b. Cyto- and receptor architectonic mapping of the human brain. In: Handbook of clinical neurology. Amsterdam: Elsevier, pp. 355–387. 10.1016/B978-0-444-63639-3.00024-4 29496153

[ref201] Passingham R. 2008. What is special about the human brain? Oxford: Oxford University Press.

[ref202] Passingham R, Stephan K, Kötter R. 2002. The anatomical basis of functional localization in the cortex. Nat Rev Neurosci. 3:606–616. 12154362 10.1038/nrn893

[ref203] Penn DC, Povinelli DJ. 2007. On the lack of evidence that non-human animals possess anything remotely resembling a ‘theory of mind’. Philos Trans R Soc B Biol Sci. 362:731–744. 10.1098/rstb.2006.2023 PMC2346530 17264056

[ref204] Petanjek Z, Judas M, Kostovic I, Uylings HBM. 2008. Lifespan alterations of basal dendritic trees of pyramidal neurons in the human prefrontal cortex: a layer-specific pattern. Cereb Cortex. 18:915–929. 17652464 10.1093/cercor/bhm124

[ref205] Petanjek Z, Judaš M, Šimić G, Rašin MR, Uylings HBM, Rakic P, Kostović I. 2011. Extraordinary neoteny of synaptic spines in the human prefrontal cortex. Proc Natl Acad Sci. 108:13281–13286. 21788513 10.1073/pnas.1105108108 PMC3156171

[ref206] Petanjek Z, Sedmak D, Džaja D, Hladnik A, Rašin MR, Jovanov-Milosevic N. 2019. The protracted maturation of associative layer IIIC pyramidal neurons in the human prefrontal cortex during childhood: a major role in cognitive development and selective alteration in autism. Front Psych. 10:122. 10.3389/fpsyt.2019.00122 PMC6426783 30923504

[ref207] Petreanu L, Mao T, Sternson SM, Svoboda K. 2009. The subcellular organization of neocortical excitatory connections. Nature. 457:1142–1145. 19151697 10.1038/nature07709 PMC2745650

[ref208] Picco N, García-Moreno F, Maini PK, Woolley TE, Molnár Z. 2018. Mathematical modeling of cortical neurogenesis reveals that the founder population does not necessarily scale with neurogenic output. Cereb Cortex. 28:2540–2550. 29688292 10.1093/cercor/bhy068 PMC5998983

[ref209] Pilley JW, Reid AK. 2011. Border collie comprehends object names as verbal referents. Behav Processes. 86:184–195. 21145379 10.1016/j.beproc.2010.11.007

[ref210] Posner MI. 2007. Evolution and development of self-regulation. James Arthur Lect. 77:1–25. 19235283

[ref211] Pradhan N, Dasgupta S, Sinha S. 2011. Modular organization enhances the robustness of attractor network dynamics. EPL Europhys Lett. 94:38004.

[ref212] Premack D, Premack A. 1996. Why animals lack pedagogy and some cultures have more of it than others. In: Olson DR, editor. The handbook of education and human development: new models of learning, teaching and schooling. Malden (MA): Blackwell Publisher.

[ref213] Premack D, Premack A. 2003. Original intelligence: unlocking the mystery of who we are. New York: McGraw-Hill.

[ref214] Premack D, Woodruff G. 1978. Does the chimpanzee have a theory of mind? Behav Brain Sci. 1:515–526.

[ref215] Purves D, Lichtman JW. 1980. Elimination of synapses in the developing nervous system. Sci N Y NY. 210:153–157. 10.1126/science.7414326 7414326

[ref216] Rakic P. 1976. Prenatal genesis of connections subserving ocular dominance in the rhesus monkey. Nature. 261:467–471. 819835 10.1038/261467a0

[ref217] Rakic P. 1988. Specification of cerebral cortical areas. Science. 241:170–176. 3291116 10.1126/science.3291116

[ref218] Rakic P. 2009. Evolution of the neocortex: a perspective from developmental biology. Nat Rev Neurosci. 10:724–735. 19763105 10.1038/nrn2719 PMC2913577

[ref219] Rakic P, Bourgeois J-P, Goldman-Rakic PS. 1994. Synaptic development of the cerebral cortex: implications for learning, memory, and mental illness. Prog Brain Res. 102:227–243. 7800815 10.1016/S0079-6123(08)60543-9

[ref220] Raman DV, Rotondo AP, O’Leary T. 2019. Fundamental bounds on learning performance in neural circuits. Proc Natl Acad Sci. 116:10537–10546. 31061133 10.1073/pnas.1813416116 PMC6535002

[ref221] Rash BG, Duque A, Morozov YM, Arellano JI, Micali N, Rakic P. 2019. Gliogenesis in the outer subventricular zone promotes enlargement and gyrification of the primate cerebrum. Proc Natl Acad Sci. 116:7089–7094. 30894491 10.1073/pnas.1822169116 PMC6452694

[ref222] Redfern PA. 1970. Neuromuscular transmission in new-born rats. J Physiol. 209:701–709. 5499804 10.1113/jphysiol.1970.sp009187 PMC1395553

[ref223] Richter CG, Coppola R, Bressler SL. 2018. Top-down beta oscillatory signaling conveys behavioral context in early visual cortex. Sci Rep. 8:6991. 29725028 10.1038/s41598-018-25267-1 PMC5934398

[ref224] Rodriguez E, George N, Lachaux J-P, Martinerie J, Renault B, Varela FJ. 1999. Perception’s shadow: long-distance synchronization of human brain activity. Nature. 397:430–433. 9989408 10.1038/17120

[ref225] Rodriguez N, Izquierdo E, Ahn Y-Y. 2019. Optimal modularity and memory capacity of neural reservoirs. Netw Neurosci. 3:551–566. 31089484 10.1162/netn_a_00082 PMC6497001

[ref226] Romer Thomsen K, Joensson M, Lou HC, Moller A, Gross J, Kringelbach ML, Changeux J-P. 2013. Altered paralimbic interaction in behavioral addiction. Proc Natl Acad Sci. 110:4744–4749. 23487797 10.1073/pnas.1302374110 PMC3606979

[ref227] Sanides F. 1962. Die Architektonik des Menschlichen Stirnhirns. Springer-Verlag Berlin Heidelberg GmbH.

[ref228] Sanides F. 1970. Functional architecture of motor and sensory cortices in primates in the light of a new concept of neocortex evolution. In: The primate brain: advances in primatology. New York: Appleton-Century-Crofts Educational Division/Meredith Corporation, pp. 137–208.

[ref229] Sanides F, Krishnamurti A. 1967. Cytoarchitectonic subdivisions of sensorimotor and prefrontal regions and of bordering insular and limbic fields in slow Loris (*Nycticebus coucang* coucang). J Hirnforsch. 9:225–252. 6077605

[ref230] Savage-Rumbaugh ES, Murphy J, Sevcik RA, Brakke KE, Williams SL, Rumbaugh DM, Bates E. 1993. Language comprehension in ape and child. Monogr Soc Res Child Dev. 58:i. 8366872

[ref231] Scannell JW, Young MP. 1993. The connectional organization of neural systems in the cat cerebral cortex. Curr Biol. 3:191–200. 15335765 10.1016/0960-9822(93)90331-h

[ref232] Schenker NM, Buxhoeveden DP, Blackmon WL, Amunts K, Zilles K, Semendeferi K. 2008. A comparative quantitative analysis of cytoarchitecture and minicolumnar organization in Broca’s area in humans and great apes. J Comp Neurol. 510:117–128. 18612968 10.1002/cne.21792

[ref233] Schenker NM, Hopkins WD, Spocter MA, Garrison AR, Stimpson CD, Erwin JM, Hof PR, Sherwood CC. 2010. Broca’s area homologue in chimpanzees (*Pan troglodytes*): probabilistic mapping, asymmetry, and comparison to humans. Cereb Cortex. 20:730–742. 19620620 10.1093/cercor/bhp138 PMC2820707

[ref234] Scholtens LH, Schmidt R, de Reus MA, van den Heuvel MP. 2014. Linking macroscale graph analytical organization to microscale neuroarchitectonics in the macaque connectome. J Neurosci. 34:12192–12205. 25186762 10.1523/JNEUROSCI.0752-14.2014 PMC6608464

[ref235] Schomers MR, Garagnani M, Pulvermüller F. 2017. Neurocomputational consequences of evolutionary connectivity changes in Perisylvian language cortex. J Neurosci. 37:3045–3055. 28193685 10.1523/JNEUROSCI.2693-16.2017 PMC5354338

[ref236] Schurger A, Sarigiannidis I, Naccache L, Sitt JD, Dehaene S. 2015. Cortical activity is more stable when sensory stimuli are consciously perceived. Proc Natl Acad Sci. 112:E2083–E2092. 25847997 10.1073/pnas.1418730112 PMC4413285

[ref237] Sedmak D, Hrvoj-Mihić B, Džaja D, Habek N, Uylings HBM, Petanjek Z. 2018. Biphasic dendritic growth of dorsolateral prefrontal cortex associative neurons and early cognitive development. Croat Med J. 59:189–202. 30394011 10.3325/cmj.2018.59.189 PMC6240825

[ref238] Semendeferi K, Armstrong E, Schleicher A, Zilles K, Hoesen GWV. 2001. Prefrontal cortex in humans and apes: a comparative study of area 10. Am J Phys Anthropol. 114:224–241. 11241188 10.1002/1096-8644(200103)114:3<224::AID-AJPA1022>3.0.CO;2-I

[ref239] Semendeferi K, Lu A, Schenker N, Damasio H. 2002. Humans and great apes share a large frontal cortex. Nat Neurosci. 5:272–276. 11850633 10.1038/nn814

[ref240] Setoh P, Scott RM, Baillargeon R. 2016. Two-and-a-half-year-olds succeed at a traditional false-belief task with reduced processing demands. Proc Natl Acad Sci. 113:13360–13365. 27821728 10.1073/pnas.1609203113 PMC5127346

[ref241] Shatz CJ, Stryker MP. 1978. Ocular dominance in layer IV of the cat’s visual cortex and the effects of monocular deprivation. J Physiol. 281:267–283. 702379 10.1113/jphysiol.1978.sp012421 PMC1282696

[ref242] Sheu S-H, Tapia JC, Tsuriel S, Lichtman JW. 2017. Similar synapse elimination motifs at successive relays in the same efferent pathway during development in mice. Elife. 6:e23193. 28157072 10.7554/eLife.23193 PMC5315461

[ref243] Shi H, Kichaev G, Pasaniuc B. 2016. Contrasting the genetic architecture of 30 complex traits from summary association data. Am J Hum Genet. 99:139–153. 27346688 10.1016/j.ajhg.2016.05.013 PMC5005444

[ref244] Shi L, Luo X, Jiang J, Chen Y, Liu C, Hu T, Li M, Lin Q, Li Y, Huang J et al. 2019. Transgenic rhesus monkeys carrying the human MCPH1 gene copies show human-like neoteny of brain development. Natl Sci Rev. 6:480–493. 10.1093/nsr/nwz043 PMC8291473 34691896

[ref245] Sjöstedt E, Zhong W, Fagerberg L, Karlsson M, Mitsios N, Adori C, Oksvold P, Edfors F, Limiszewska A, Hikmet F et al. 2020. An atlas of the protein-coding genes in the human, pig, and mouse brain. Science. 367:eaay5947. 32139519 10.1126/science.aay5947

[ref246] Smith K, Bastin ME, Cox SR, Valdés Hernández MC, Wiseman S, Escudero J, Sudlow C. 2019. Hierarchical complexity of the adult human structural connectome. Neuroimage. 191:205–215. 30772400 10.1016/j.neuroimage.2019.02.028 PMC6503942

[ref247] Somel M, Liu X, Khaitovich P. 2013. Human brain evolution: transcripts, metabolites and their regulators. Nat Rev Neurosci. 14:112–127. 23324662 10.1038/nrn3372

[ref248] Sporns O. 2006. Small-world connectivity, motif composition, and complexity of fractal neuronal connections. Biosystems. 85:55–64. 16757100 10.1016/j.biosystems.2006.02.008

[ref249] Stepanyants A, Hof PR, Chklovskii DB. 2002. Geometry and structural plasticity of synaptic connectivity. Neuron. 34:275–288. 11970869 10.1016/s0896-6273(02)00652-9

[ref250] Striedter GF. 2005. Principles of brain evolution. Sunderland (MA): Sinauer Associates.

[ref251] Suzuki IK. 2020. Molecular drivers of human cerebral cortical evolution. Neurosci Res. 151:1–14. 31175883 10.1016/j.neures.2019.05.007

[ref252] Suzuki IK, Gacquer D, Van Heurck R, Kumar D, Wojno M, Bilheu A, Herpoel A, Lambert N, Cheron J, Polleux F et al. 2018. Human-specific NOTCH2NL genes expand cortical neurogenesis through Delta/NOTCH regulation. Cell. 173:1370–1384.e16. 29856955 10.1016/j.cell.2018.03.067 PMC6092419

[ref253] Suzuki M, Larkum ME. 2020. General anesthesia decouples cortical pyramidal neurons. Cell. 180:666–676.e13. 32084339 10.1016/j.cell.2020.01.024

[ref254] The Chimpanzee Sequencing and Analysis Consortium. 2005. Initial sequence of the chimpanzee genome and comparison with the human genome. Nature. 437:69–87. 16136131 10.1038/nature04072

[ref255] Tomasello R, Garagnani M, Wennekers T, Pulvermüller F. 2018. A neurobiologically constrained cortex model of semantic grounding with spiking neurons and brain-like connectivity. Front Comput Neurosci. 12:88. 30459584 10.3389/fncom.2018.00088 PMC6232424

[ref256] Tononi G, Boly M, Massimini M, Koch C. 2016. Integrated information theory: from consciousness to its physical substrate. Nat Rev Neurosci. 17:450–461. 27225071 10.1038/nrn.2016.44

[ref257] Tononi G, Edelman GM. 1998. Consciousness and complexity. Science. 282:1846–1851. 9836628 10.1126/science.282.5395.1846

[ref258] Tononi G, Sporns O, Edelman GM. 1999. Measures of degeneracy and redundancy in biological networks. Proc Natl Acad Sci U S A. 96:3257–3262. 10077671 10.1073/pnas.96.6.3257 PMC15929

[ref259] Tsigelny IF, Kouznetsova VL, Baitaluk M, Changeux J-P. 2013. A hierarchical coherent-gene-group model for brain development: a hierarchical gene groups model for brain development. Genes Brain Behav. 12:147–165. 23173912 10.1111/gbb.12005

[ref260] Turney SG, Walsh MK, Lichtman JW. 2012. In vivo imaging of the developing neuromuscular junction in neonatal mice. Cold Spring Harb Protoc. 2012:1166–1176. 23118362 10.1101/pdb.prot072082

[ref261] Uhlhaas PJ, Singer W. 2011. The development of neural synchrony and large-scale cortical networks during adolescence: relevance for the pathophysiology of schizophrenia and neurodevelopmental hypothesis. Schizophr Bull. 37:514–523. 21505118 10.1093/schbul/sbr034 PMC3080681

[ref262] Uttal WR. 2001. The new phrenology: the limits of localizing cognitive processes in the brain, Life and mind: philosophical issues in biology and psychology. Cambridge (MA): MIT Press.

[ref263] Vallender EJ. 2014. Bringing non-human primate research into the post-genomic era: how monkeys are teaching us about elite controllers of HIV/AIDS. Genome Biol. 15:507. 25417723 10.1186/s13059-014-0507-y PMC4282011

[ref264] van den Heuvel MP, Sporns O. 2011. Rich-club organization of the human connectome. J Neurosci. 31:15775–15786. 22049421 10.1523/JNEUROSCI.3539-11.2011 PMC6623027

[ref265] van Dyck LI, Morrow EM. 2017. Genetic control of postnatal human brain growth. Curr Opin Neurol. 30:114–124. 27898583 10.1097/WCO.0000000000000405 PMC5340196

[ref266] Van Essen DC, Glasser MF, Dierker DL, Harwell J. 2012. Cortical parcellations of the macaque monkey analyzed on surface-based atlases. Cereb Cortex. 22:2227–2240. 22052704 10.1093/cercor/bhr290 PMC3500860

[ref267] Vince G. 2019. Transcendence: how humans evolved through fire, language, beauty, and time. London, UK: Allen Lane.

[ref268] von Economo C, Koskinas GN. 1925. Die Cytoarchitektonik der Hirnrinde des erwachsenen Menschen. Berlin: Springer.

[ref269] Vygotskiĭ LS, Cole M. 1978. Mind in society: the development of higher psychological processes. Cambridge (MA): Harvard University Press.

[ref270] Vyshedskiy A. 2019. Language evolution to revolution: the leap from rich-vocabulary non-recursive communication system to recursive language 70,000 years ago was associated with acquisition of a novel component of imagination, called prefrontal synthesis, enabled by a mutation that slowed down the prefrontal cortex maturation simultaneously in two or more children – the Romulus and Remus hypothesis. Res Ideas Outcomes. 5:e38546.

[ref271] Waddington CH. 1942. The epigenotype. Endeavour. 1:18–20. (reprinted in International Journal of Epidemiology 2012;41:10–13). 10.1093/ije/dyr184 22186258

[ref272] Wagstyl K, Ronan L, Goodyer IM, Fletcher PC. 2015. Cortical thickness gradients in structural hierarchies. Neuroimage. 111:241–250. 25725468 10.1016/j.neuroimage.2015.02.036 PMC4401442

[ref273] Wang P, Kong R, Kong X, Liégeois R, Orban C, Deco G. 2019. Inversion of a large-scale circuit model reveals a cortical hierarchy in the dynamic resting human brain. Sci Adv. 5:eaat7854. 10.1126/sciadv.aat7854 PMC6326747 30662942

[ref274] Wang S-J, Hilgetag CC, Zhou C. 2011. Sustained activity in hierarchical modular neural networks: self-organized criticality and oscillations. Front Comput Neurosci. 5:30. 21852971 10.3389/fncom.2011.00030 PMC3151620

[ref275] Wang X-J. 2002. Probabilistic decision making by slow reverberation in cortical circuits. Neuron. 36:955–968. 12467598 10.1016/s0896-6273(02)01092-9

[ref276] Wang X-J. 2008. Decision making in recurrent neuronal circuits. Neuron. 60:215–234. 18957215 10.1016/j.neuron.2008.09.034 PMC2710297

[ref277] Wang X-J, Tegner J, Constantinidis C, Goldman-Rakic PS. 2004. Division of labor among distinct subtypes of inhibitory neurons in a cortical microcircuit of working memory. Proc Natl Acad Sci. 101:1368–1373. 14742867 10.1073/pnas.0305337101 PMC337059

[ref278] Weyer S, Pääbo S. 2016. Functional analyses of transcription factor binding sites that differ between present-day and archaic humans. Mol Biol Evol. 33:316–322. 26454764 10.1093/molbev/msv215 PMC4866544

[ref279] Wiesel TN, Hubel DH. 1963. Single-cell responses in striate cortex of kittens deprived of vision in one eye. J Neurophysiol. 26:1003–1017. 14084161 10.1152/jn.1963.26.6.1003

[ref280] Wu X, Fu Y, Knott G, Lu J, Di Cristo G, Huang ZJ. 2012. GABA signaling promotes synapse elimination and axon pruning in developing cortical inhibitory interneurons. J Neurosci. 32:331–343. 22219294 10.1523/JNEUROSCI.3189-11.2012 PMC3742883

[ref281] Wyss R, König P, Verschure PFMJ. 2006. A model of the ventral visual system based on temporal stability and local memory. PLoS Biol. 4:e120. 16605306 10.1371/journal.pbio.0040120 PMC1436026

[ref282] Xie H, Liu Y, Zhu Y, Ding X, Yang Y, Guan J-S. 2014. In vivo imaging of immediate early gene expression reveals layer-specific memory traces in the mammalian brain. Proc Natl Acad Sci. 111:2788–2793. 24550309 10.1073/pnas.1316808111 PMC3932903

[ref283] Zelazo PD. 2004. The development of conscious control in childhood. Trends Cogn Sci. 8:12–17. 14697398 10.1016/j.tics.2003.11.001

[ref284] Zembrzycki A, Stocker AM, Leingärtner A, Sahara S, Chou S-J, Kalatsky V, May SR, Stryker MP, O’Leary DD. 2015. Genetic mechanisms control the linear scaling between related cortical primary and higher order sensory areas. Elife. 4:e11416. 26705332 10.7554/eLife.11416 PMC4739755

[ref285] Zeng H, Shen EH, Hohmann JG, Oh SW, Bernard A, Royall JJ, Glattfelder KJ, Sunkin SM, Morris JA, Guillozet-Bongaarts AL et al. 2012. Large-scale cellular-resolution gene profiling in human neocortex reveals species-specific molecular signatures. Cell. 149:483–496. 22500809 10.1016/j.cell.2012.02.052 PMC3328777

[ref286] Zilles K. 2002. Architectonics of the human cerebral cortex and transmitter receptor fingerprints: reconciling functional neuroanatomy and neurochemistry. Eur Neuropsychopharmacol. 12:587–599. 12468022 10.1016/s0924-977x(02)00108-6

[ref287] Zilles K. 2005. Evolution of the human brain and comparative cyto-and receptor architecture. In: From monkey brain to human brain. Cambridge, MA: MIT Press, pp. 41–56.

[ref288] Zilles K, Palomero-Gallagher N. 2017. Multiple transmitter receptors in regions and layers of the human cerebral cortex. Front Neuroanat. 11:78. 10.3389/fnana.2017.00078 PMC5609104 28970785

